# Mitochondrial defects in the respiratory complex I contribute to impaired translational initiation via ROS and energy homeostasis in SMA motor neurons

**DOI:** 10.1186/s40478-020-01101-6

**Published:** 2020-12-22

**Authors:** Maximilian Paul Thelen, Brunhilde Wirth, Min Jeong Kye

**Affiliations:** 1 Institute of Human Genetics, University of Cologne, Kerpener Str. 34, 50931 Cologne, Germany; 2 Center for Molecular Medicine, Cologne, University of Cologne, 50931 Cologne, Germany; 3 Center for Rare Diseases Cologne, University Hospital Cologne, University of Cologne, 50931 Cologne, Germany

**Keywords:** Spinal muscular atrophy, SMN, *SMN1*, *SMN2*, Mitochondria, Reactive oxygen species, Translation initiation

## Abstract

Spinal muscular atrophy (SMA) is a neuromuscular disease characterized by loss of lower motor neurons, which leads to proximal muscle weakness and atrophy. SMA is caused by reduced survival motor neuron (SMN) protein levels due to biallelic deletions or mutations in the *SMN1* gene. When SMN levels fall under a certain threshold, a plethora of cellular pathways are disturbed, including RNA processing, protein synthesis, metabolic defects, and mitochondrial function. Dysfunctional mitochondria can harm cells by decreased ATP production and increased oxidative stress due to elevated cellular levels of reactive oxygen species (ROS). Since neurons mainly produce energy via mitochondrial oxidative phosphorylation, restoring metabolic/oxidative homeostasis might rescue SMA pathology. Here, we report, based on proteome analysis, that SMA motor neurons show disturbed energy homeostasis due to dysfunction of mitochondrial complex I. This results in a lower basal ATP concentration and higher ROS production that causes an increase of protein carbonylation and impaired protein synthesis in SMA motor neurons. Counteracting these cellular impairments with pyruvate reduces elevated ROS levels, increases ATP and SMN protein levels in SMA motor neurons. Furthermore, we found that pyruvate-mediated SMN protein synthesis is mTOR-dependent. Most importantly, we showed that ROS regulates protein synthesis at the translational initiation step, which is impaired in SMA. As many neuropathies share pathological phenotypes such as dysfunctional mitochondria, excessive ROS, and impaired protein synthesis, our findings suggest new molecular interactions among these pathways. Additionally, counteracting these impairments by reducing ROS and increasing ATP might be beneficial for motor neuron survival in SMA patients.

## Background

Spinal muscular atrophy (SMA) is an inherited neuromuscular disease that is characterized by loss of lower motor neurons (MNs) due to reduced levels of the ubiquitously expressed survival motor neuron (SMN) protein [1]. The incidence of SMA varies between 1 per 6000–10,000 newborns in the human population [2]. In 96% of SMA patients, homozygous deletions, or mutations in *SMN1*, the gene encoding for full-length SMN, have been described [2]. Interestingly, the human genome contains an almost identical gene - *SMN2* -, that mainly produces a transcript lacking exon 7 due to a single silent mutation [3]. Hence, *SMN2* produces approximately 10% of the full-length SMN protein compared to *SMN1.* As the amount of SMN protein inversely correlates with disease severity [4], the copy number of *SMN2* is often used as a good predictor of SMA severity. SMN forms a stable complex with other proteins such as Gemin2-8 [5]. Additionally, SMN is an RNA binding protein that is involved in multiple essential cellular functions, including biogenesis of spliceosomal small nuclear ribonucleoproteins (snRNPs) [6] and trafficking of mRNAs to axon terminals [7–10]. Furthermore, SMN deficient cells show dysregulated splicing and miRNA processing [11, 12], hyperexcitability and impaired Ca^2+^ homeostasis [13–15], decreased translation [8, 16, 17], and also results in impaired axon growth [9]. These findings suggest SMN as a multifunctional protein.

Recent evidence suggests that energy metabolism is impaired in SMA, including mitochondria and glucose metabolism [18–20]. In the mitochondrial oxidative phosphorylation (OXPHOS), the NADH:ubiquinone oxidoreductase (complex I) of the electron transport chain is the rate-limiting enzyme in respiration and the major producer of reactive oxygen species (ROS) [21, 22]. Therefore, a dysfunctional complex I could impair energy production as well as ROS homeostasis. ROS can function as signaling molecules by activating various pathways, including MAPK, PI3K, and Ca^2+^ signaling [21], and harm cells by irreversible protein modifications such as carbonylation [23, 24]. For example, oxidative stress can hinder the SMN complex formation [25].

Various cellular mechanisms regulate neuronal protein synthesis, including trafficking of molecules, axonal local translation, and local protein degradation. Notably, impaired protein synthesis has been reported in various neurodegenerative disorders, including Alzheimer’s disease (AD) and SMA. The mammalian target of rapamycin (mTOR) is the master regulator of protein synthesis in neurons. Lost balance in the mTOR pathway causes neuronal dysfunction from Tuberous sclerosis complex (TSC) to Rett syndrome [26]. mTOR can form two complexes based on its binding partners, mTORC1 and mTORC2. mTORC1 acts as an integral node between cellular energy production and consumption. It promotes anabolic processes but also restricts catabolic processes such as autophagy [27–29]. Anabolic processes promoted by active mTORC1 include mitochondrial biogenesis [30] and protein synthesis [31]. Cap-dependent translation initiation is promoted by mTORC1 via phosphorylation of 4E-BP1 or S6 kinase (S6K) [32–34].

Here, we first report the molecular mechanism explaining how mitochondria regulate protein synthesis via ATP and ROS production in spinal MNs. Furthermore, when this pathway is disturbed, it contributes to the MN disease, SMA. Particularly, cellular ATP and ROS signaling regulate protein synthesis at the translational initiation step, which is impaired in SMA MNs. Our findings imply that mitochondria control neuronal energy and ROS homeostasis, and protein synthesis via the mTOR pathway.

## Materials and methods

### Animal model

An SMA mouse model carrying two *SMN2* copies on one allele and a murine *Smn* null, FVB/N background was used [35]. It was established by breeding *Smn*^KO/KO^; *SMN2*^tg/tg^ mice with *Smn*^KO/WT^ mice resulting in 50% SMA mice (*Smn*^KO/KO^; *SMN2*^tg/0^) and 50% phenotypically normal heterozygous littermates (*Smn*^KO/WT^; *SMN2*^tg/0^) [36]. FVB/N wild type mice were used as controls (Charles River).

### Primary MN culture

E13.5 embryos were used for primary MN culture. SMA embryos were genotyped (KAPA mouse genotyping kit, Sigma) and cultured as previously described [36]. Briefly, spinal cords were isolated and dissociated with 1% trypsin (Worthington) in PBS. Single cell suspension was achieved by trituration with DNase I (Applichem) in plating medium (Dulbecco’s Modified Eagle’s Medium (DMEM) with 5% fetal calf serum (Biochrom), 0.6% glucose, penicillin/streptomycin (Thermo Fisher Scientific), and amphotericin B (Promocell)). For imaging analyses, 15,000 cells/cm^2^ were plated on poly-d-lysine (PDL, 10 µg/ml, Sigma) coated coverslips, and for biochemical analyses, 120,000 cells/cm^2^ were plated on PDL coated plates with plating media. Plating media was replaced by MN maintenance medium (Neurobasal medium with B27 supplement (Thermo Fisher Scientific), 2 mM l-glutamine, 1% penicillin/streptomycin and 0.25% amphotericin B with additional growth factors: brain derived neurotrophic factor (BDNF, 10 ng/ml), ciliary neurotrophic factor (CNTF, 10 ng/ml), and glia cell line derived neurotrophic factor (GDNF, 10 ng/ml, all purchased from PeproTech)). Half of the medium was exchanged every third day, and cytosine arabinoside (AraC) was added continuously after 3 days to a final concentration of 1 μM. Cells were cultured at 37 °C in a humidified incubator with 5% CO_2_.

### Culture of cell lines

MN-like NSC-34 [37] cells were cultured in DMEM with 10% fetal calf serum, 1% penicillin/streptomycin, and amphotericin B. 20,000 cells/cm^2^ were plated onto PDL coated 12- well plates. Differentiation was induced by 50 µM retinoic acid (Sigma) for 3 days. Cells were maintained at 37 °C in a humidified incubator with 5% CO_2_.

### RNA isolation, cDNA synthesis and real-time PCR

Total RNA was extracted from cells using the mirVana™ miRNA Isolation Kit (Thermo Fisher Scientific) according to the manufacturer’s instructions. cDNA was produced from total RNA using the High-Capacity cDNA Reverse Transcription Kit (Thermo Fisher Scientific) with random primers. mRNA expression levels were quantified with PowerSYBR^®^ Green PCR Master Mix (Thermo Fisher Scientific) and 1 μM of gene-specific primers using real-time PCR (7500 Real-Time PCR System, Thermo Fisher Scientific). Specificity of the primers was confirmed by Sanger’s sequencing. Sequences of primers are listed in Additional file 1: Supplementary Table 1.

### Protein isolation and Western blot analysis

Proteins were extracted with RIPA buffer (Sigma) with protease and phosphatase inhibitors (Thermo Fisher Scientific), and protein concentration was determined by Pierce™ BCA protein assay kit (Thermo Fisher Scientific). Western blot analysis was performed with a standard protocol. The information about antibodies is listed in Additional file 1: Supplementary Table 2. Signals were detected with ChemiDoc XRS + System (BioRad) using ECL chemiluminescence (Thermo Scientific) and quantified using the ImageLab 6.0 software (BioRad).

### MitoTracker^®^ and immunostaining of MNs

Cells were incubated with MitoTracker Red CMXRos (Thermo Fisher Scientific) at a final concentration of 100 nM for 15 min and fixed with 4% paraformaldehyde (PFA). The information about dyes can be found in Additional file 1: Supplementary Table 3. Images were acquired with a fluorescence microscope (Zeiss Axio Imager.M2) equipped with an AxioCam MR camera and an ApoTome.2 system and analyzed with FIJI. All image analyses were performed blindly.

### Drug treatment

Primary MNs and NSC34 cells were supplemented in culture with the following substances at given concentrations in each figure legend and Additional file 1: Supplementary Table 4: The supplements sodium pyruvate and sodium lactate, the antioxidant N-acetylcysteine (NAC), the ROS inducer menadione, the protein synthesis inhibitor anisomycin (all purchased from Sigma) and the water-soluble mTOR inhibitor WYE-687 dihydrochloride (Tocris).

### Proteomics of primary MNs

Primary MNs were treated with 50 mM pyruvate, 10 µM NAC, and 100 µM menadione for 1 h, lysed in RIPA buffer with protease and phosphatase inhibitors, and further processed for mass spectrometry (MS) analysis. Proteins were precipitated using ice-cold acetone at −80 °C for 15 min and at − 20 °C for 2 h. Next, proteins were resuspended in 8 M urea buffer with protease inhibitor, reduced in-solution using 5 mM dithiothreitol (DTT) at room temperature (RT) for 1 h, alkylated with 40 mM chloroacetamide (CAA) in the dark for 30 min, and digested with endo-proteinase Lys-C at RT for 4 h. Samples were diluted with 50 mM triethylammoniumbicarbonate (TEAB) to a final concentration of urea ≤ 2 M and digested with trypsin at RT for 8 h. After digestion was completed, samples were acidified with 1% formic acid and purified with styrenedivinylbenzene-reverse phase sulfonate (SDB-RPS) Stage Tips. Proteomic analysis was performed with ultra-high-performance liquid chromatography (UHPLC) coupled to a Quadrupole-Orbitrap mass spectrometer (CECAD/CMMC Proteomics core facility, University of Cologne). Raw data were analyzed by MaxQuant. As a reference, a canonical mouse database from Uniprot (22.08.19) was used. For gene ontology (GO) analysis, the Database for Annotation, Visualization and Integrated Discovery 6.7 (DAVID) was used. Statistical analysis and data visualization were performed using RStudio and R version 3.6.2. We used the mouse MitoCarta2.0 dataset to identify mitochondrial proteins [38]. Additional data about identified proteins and GO analysis are listed in Additional file 2: MS Source data.

### Isolation of mitochondria and complex I activity assay

Mitochondria were isolated from cultured primary MNs using the Mitochondria Isolation Kit (Abcam) according to the manufacturer’s instructions. In brief, 3 × 10^7^ 10 days in vitro (DIV) cultured MNs were collected, frozen for 10 min, and subsequently thawed. Cells were disrupted by a pre-cooled Dounce homogenizer after 10 min incubation on ice. After additional centrifugation at 1000 x g, the supernatant was collected and centrifuged again at 12,000 x g. Finally, isolated mitochondria were resuspended in a storage buffer and kept at −80 °C until further use. To test the functionality of mitochondrial respiratory chain complexes, complex I enzyme activity assay kit (Abcam) was used. In brief, proteins extracted from the isolated mitochondria were loaded onto a microwell plate and complex I proteins were attached to the microplate via antibody coupling. Complex I activity was measured by the optical density (OD_450_ nm) after adding substrates. The enzyme kinetic was followed for 1 h. We used 300 µg of cell extracts for tissue samples from heart and 20 µg of mitochondrial extracts for MN samples.

### SUnSET assay (surface sensing of translation)

Protein synthesis was measured by SUnSET assay [39]. Cells were treated with ROS modifying drugs or 50 µM anisomycin as negative control. Afterward, the cells were incubated with 1 µM puromycin for 30 min. Finally, puromycin labeled peptides were detected by western blotting or immunostaining using an anti-puromycin antibody. Axons were selected with a segmented line, straightened, and divided into 20 µm bins using the concentric circles plugin. Mean puromycin intensities were quantified using plot profiles.

### SunRiSE (SUnSET-based Ribosome Speed of Elongation)

SunRiSE assay was used to monitor protein elongation speed [40]. Initiation of mRNA translation was blocked by 2 µg/ml harringtonine (Abcam) at different time points, and all samples were incubated with 10 µg/ml (16.7 µM) puromycin for 10 min. Puromycin labeled peptides were detected by western blotting.

### ATP assay

Intracellular ATP concentrations were measured with the ATP determination kit (Thermo Fisher Scientific) based on bioluminescence signal detection by the GloMax^®^ luminescence reader (Promega). ATP levels were normalized to the amounts of soluble proteins determined by BCA assay.

### Glucose uptake assay

Glucose Uptake-Glo kit (Promega) was used to measure glucose uptake efficiency using 120,000 cells/cm^2^. We mainly followed the manufacturer’s instruction and luminescence was detected by the GloMax luminescence reader. Signal was normalized to protein concentration measured by BCA assay.

### Pyruvate uptake assay

Pyruvate uptake was measured with the Pyruvate assay kit (Sigma). Fluorescence signal was measured with a Safire 2 microplate reader (Tecan) and normalized to soluble protein amount.

### Detection of oxidative stress

CellROX^®^ Green reagent (Thermo Fisher Scientific) was used to measure oxidative stress. Cells were incubated with 5 µM CellROX Green reagent for 30 min, fixed with 3.7% formaldehyde, nuclei, and F-actin were labeled with DAPI and Alexa Fluor 568 Phalloidin (Thermo Fisher Scientific), respectively. Images were acquired with a fluorescence microscope equipped with an ApoTome.2 system. CellROX signal was quantified with a Safire 2 microplate reader (Tecan). Signals of the microplate reader were normalized to soluble protein concentration after lysis with RIPA buffer.

### Protein carbonylation assay

The amount of protein carbonyl groups was measured with the Protein carbonyl assay kit (Abcam). Proteins were quantified by Bradford.

### Click-iT^®^ AHA assay

To deplete endogenous methionine reserves, cells were incubated with HBSS for 30 min. 500 µM L-azidohomoalanine (AHA, Thermo Fisher Scientific) was treated to cells at 37 °C for 1 h. Cells were washed and lysed with RIPA buffer supplemented with protease and phosphatase inhibitors on ice. Click chemistry reaction was performed with Click-iT protein reaction buffer kit (Thermo Fisher Scientific), using manufacturer’s protocol.

### Statistical analysis

Statistical analysis was conducted in R version 3.6.2 using RStudio [41]. All graphs for cell biological experiments are presented as mean ± S.D. For normally distributed variables, statistical significance was analyzed with an unpaired, two-tailed Student’s t-test. Multiple comparisons are corrected with the Holm-Bonferroni method. Differences among group means are determined with Tukey’s honestly significant difference (HSD) test after rejection of the null hypothesis by one-way analysis of variance (ANOVA). Normally distributed experimental results with two factors are analyzed by two-way ANOVA with Tukey HSD post hoc analysis. To compare multiple mean values to the mean from a single control, especially for time-course and dose–response experiments, Dunnett’s post hoc test was used after one-way ANOVA. Statistical tests were applied only to biological replicates, even when data is presented as individual measurement counts. Statistical methods are listed in the figure legends of individual experiments. Significance is indicated by stars (*p < 0.05; **p < 0.01; *** < 0.001, n.s. = not significant).

## Results

### Mitochondria are defective in SMA MNs

**Fig. 1:**
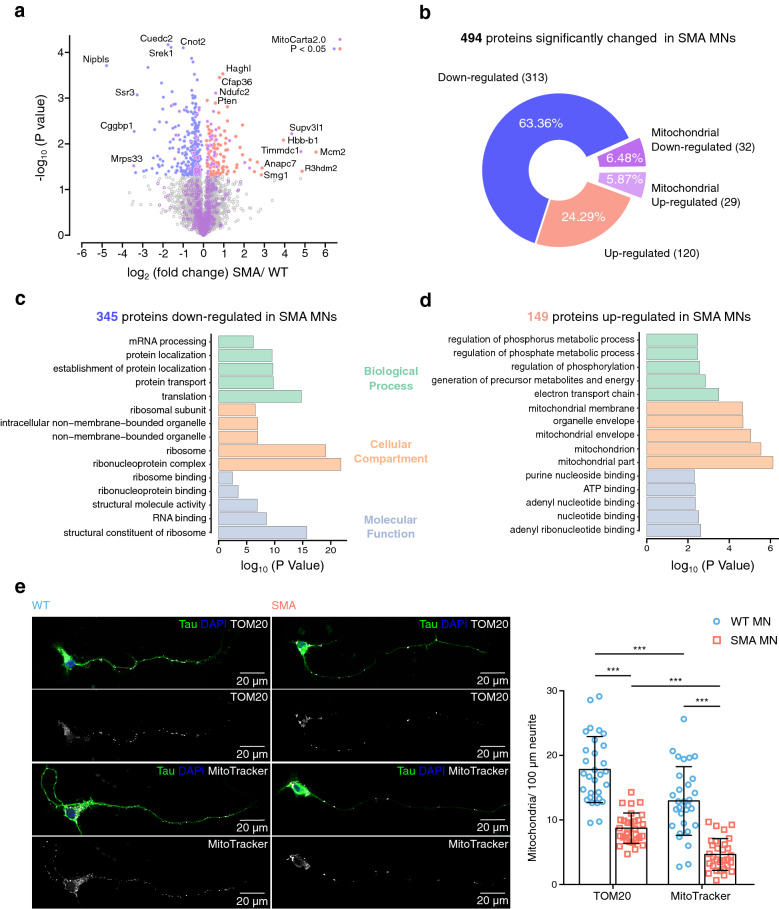
Mitochondria are defective in SMA MNs. **a**, **b** Volcano plot (**a**) and pie chart (**b**) of whole proteome analysis comparing WT and SMA MNs; plotted *p* values (−log_10_) against fold changes (log_2_, SMA/WT). Four independent samples of WT MNs and three independent samples of SMA MNs were used for analysis. *p* values were determined using unpaired two-sided t-test. Proteins with *p* < 0.05 are highlighted in blue (313 down-regulated) and red (120 up-regulated), and proteins with localization in mitochondria are marked in purple (32 down-regulated and 29 up-regulated). **c**, **d** Gene ontology (GO) analysis of 345 down-regulated (**c**) and 149 up-regulated proteins (**d**) in SMA MNs. The 5 most significant terms of each category are shown. **e** Representative images and quantification of mitochondria in 100 μm long primary axons of WT and SMA MNs labeled with anti-Tau antibody (green), DAPI (blue), and anti-TOM20 antibody or MitoTracker (white). Scale bars: 20 µm. Each dot represents the average number of mitochondria in each neuron (n = 30; biological replicates N = 3). Two-way ANOVA with Tukey HSD post hoc analysis was used to determine statistical significance for multiple comparisons. Bar graphs depict the mean ± S.D. ***p < 0.001

To discover dysregulated pathways in SMA, we investigated differentially expressed proteins in primary MNs isolated from an SMA mouse model compared to wild-type mice [35]. It has been shown that around 8101 proteins are expressed in MNs from E12.5 mouse embryos [42]. We detected 5165 proteins using whole proteome analysis of 10DIV-cultured MNs, isolated from E13.5 embryos (Fig. 1a). 494 proteins are significantly changed in SMA MNs compared to WT MNs, and 61 proteins are localized to mitochondria based on the MitoCarta2.0 database containing 1158 proteins (Fig. 1b and Additional file 3: Figure S1a). From 44 known proteins of the respiratory complex I, we identified 35 proteins in our dataset, and 9 of them were significantly altered in SMA MNs (Additional file 3: Figure S1a, S2a). To understand the biological meaning of SMA affected proteins, we performed gene ontology (GO) analysis of 345 significantly down-regulated and 149 significantly up-regulated proteins in SMA (Fig. 1c and d). Among the down-regulated proteins, we identified previously reported dysfunctional processes in SMA such as mRNA processing, protein transport, and protein synthesis, confirming the reliability of the data set (Fig. 1c) [6–9, 16]. Data from the 149 up-regulated proteins strongly suggested mitochondrial dysfunction (Fig. 1d). Therefore, we further investigated the function and localization of mitochondria in MNs by staining with MitoTracker (functional mitochondria) and TOM20 (total mitochondria). Indeed, numbers of total and functional mitochondria are reduced in SMA axons (Fig. 1e). The finding of mitochondrial mislocalization is strengthened by reduced levels of mitochondrial motor proteins KIF1B and KIF1BP in SMA MNs in our whole proteome analysis (Additional file 3: Figure S1a and Additional file 2: MS source data). In addition, we found that mitochondria are smaller and fragmented in SMA MNs (Additional file 3: Figure S1b). Together, these results suggest that mitochondria are defective in SMA MNs.

### Defective complex I can induce higher ROS levels and lower ATP levels in SMA MNs

**Fig. 2:**
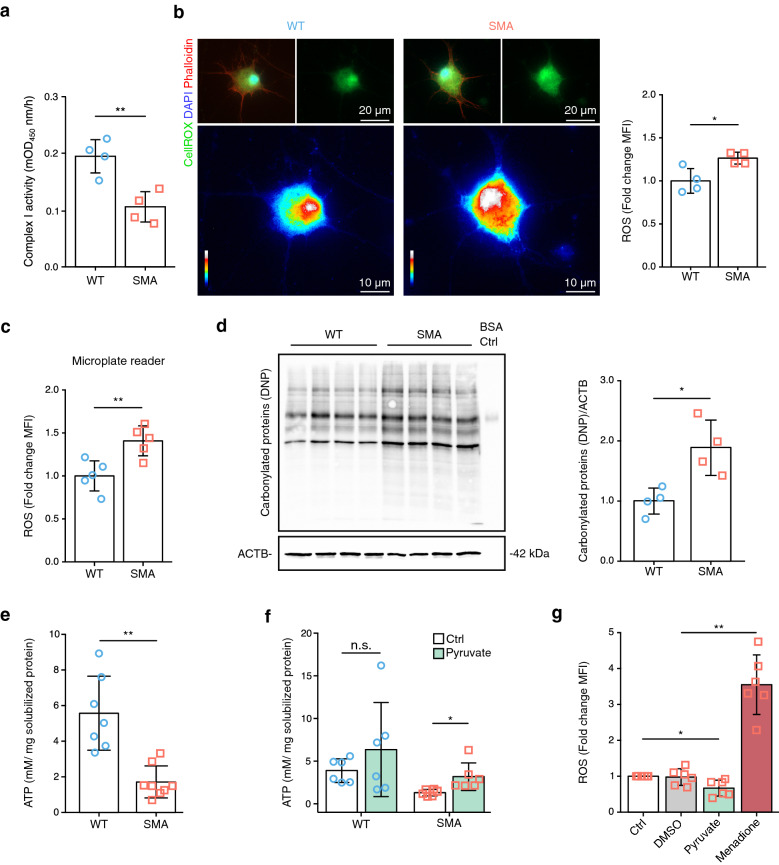
Defective mitochondrial complex I causes cellular dysfunction in SMA MNs. **a** Complex I activity using 20 µg mitochondria extract isolated from WT and SMA MNs (10DIV) (N = 4). Quantification represents the increase in mean OD_450_ nm/h. **b** Representative images and quantification of WT and SMA MNs labeled with CellROX (green and rainbow color) for ROS detection, DAPI (blue), and Phalloidin (red). Rainbow color indicates the intensity of CellROX signal. Quantification of mean fluorescence intensity of the CellROX signal in WT and SMA MN (red squares) (n = 40, N = 4). Scale bars are written on individual images. **c** Quantification of CellROX signal of WT and SMA MNs measured with a microplate reader (N = 5). **d** Western blot analysis and quantification of carbonylated proteins (DNP = 2,4-dinitrophenyl) in 10DIV WT and SMA MNs (N = 4). Bar graph represents the quantification of the DNP signal. ACTB was used as a loading control. **e** ATP levels in WT (N = 7) and SMA MNs (N = 8). Data are normalized to solubilized protein amounts. **f** ATP levels in WT and SMA MNs (N = 6) after 50 mM pyruvate treatment for 1 h. Data are normalized to solubilized protein amounts. Individual data (circle or square) represent biological replicates. **g** ROS levels of SMA MNs after 50 mM pyruvate or 100 µM menadione treatment for 1 h. Two-tailed unpaired t-tests with Holm-Bonferroni correction for multiple comparisons were used on independent biological replicates (N = 6) to determine statistical significance. *p < 0.05, **p < 0.01. **a–f** Blue circles represent data from WT and red squares represent SMA MNs. 10DIV MNs were used. Each dot represents the quantification of individual biological replicates. A two-tailed unpaired t-test was used on independent biological replicates to determine statistical significance. Bar graphs depict the mean ± S.D., n.s. *p* > 0.05, *p < 0.05, **p < 0.01

As our proteomics results indicated defects in complex I of the electron transport chain, we biochemically measured the activity of complex I in WT and SMA MNs (Additional file 3: Figure S2a and S2b). Indeed, complex I activity was 50% lower in SMA MNs (Fig. 2a and Additional file 3: Figure S2c). Additionally, we measured complex I activity in the heart, another metabolically active tissue, and found no difference between WT and SMA MNs (Additional file 3: Figure S2d). This data suggested that complex I dysfunction in SMA is MN specific. Next, as complex I is a known source of reactive oxygen species (ROS) in mitochondria [22], we measured intracellular ROS levels using CellROX. As expected, ROS levels were increased in SMA MNs compared to WT ones (Fig. 2b and Fig. 2c). Furthermore, as excessive ROS can cause carbonylation of proteins [43], we measured the levels of carbonylated proteins in SMA MNs. Indeed, higher amounts of proteins were carbonylated in SMA (Fig. 2d). These data confirmed our finding that SMA MNs are under oxidative stress.

Since mitochondrial OXPHOS besides the tricarboxylic acid (TCA) cycle is the main source of energy in neurons, we measured the intracellular ATP concentrations in WT and SMA MNs. ATP concentration is up to threefold lower in SMA compared to WT MNs (Fig. 2e). Furthermore, as ATP can also be produced by glycolysis to compensate for high energy demand in neurons [44], glucose uptake was monitored. Interestingly, glucose uptake is also impaired in SMA MNs (Additional file 3: Figure S2e). These data indicate that SMA MNs are in an energy-deprived status. Next, we pursued to restore the effects of defective mitochondria in SMA. Since pyruvate is known to reduce ROS in a non-enzymatic way and is also a known substrate of the TCA cycle [45], we supplemented MNs with sodium pyruvate. First, we confirmed that MNs can take up pyruvate within 5 min after treatment (Additional file 3: Figure S2f). While 10 mM pyruvate showed no clear effect, 50 mM pyruvate treatment for 1 h could increase ATP concentration significantly in SMA MNs (Fig. 2f and Additional file 3: Figure S2 g). However, lactate, which can be converted to pyruvate by lactate dehydrogenase in the cytoplasm [46], did not alter ATP levels in primary MNs (Additional file 3: Figure S2g). In addition, as pyruvate has been suggested to be a ROS scavenger [47], we treated SMA MNs with pyruvate and subsequently measured ROS levels. Indeed, pyruvate could successfully reduce ROS levels in SMA MNs (Fig. 2g). Further, we confirmed ROS reduction by treatment with 50 mM pyruvate or 10 µM of the antioxidant NAC in menadione mediated ROS induced NSC-34 cells (Additional file 3: Figure S2h). These results suggest that pyruvate is a valuable supplement to restore ATP levels and simultaneously balance intracellular ROS levels in MNs.

### Effect of ROS on protein synthesis in neurons

**Fig. 3:**
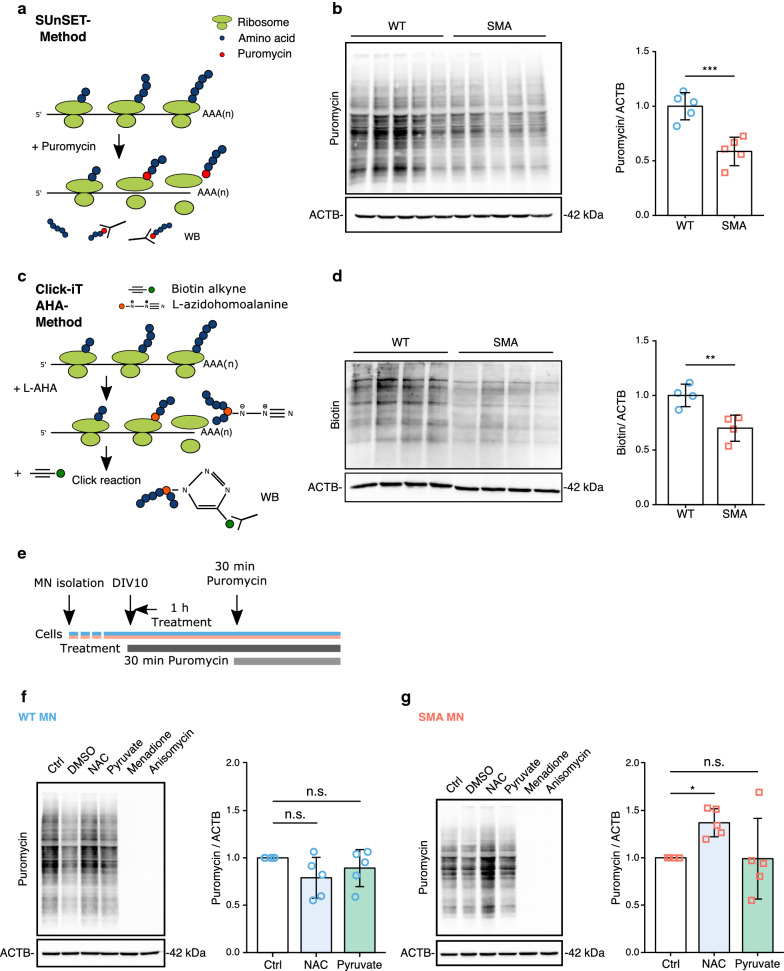
Effect of ROS on protein synthesis in neurons. **a** Schematic drawing of the SUnSET method. Puromycin labels newly synthesized proteins. Puromycin is detected by anti-puromycin antibody. **b** SUnSET assay in WT and SMA MNs (N = 5). ACTB was used as loading control. **c** Schematic drawing of the Click-iT AHA method. L-azidohomoalanine (L-AHA) is incorporated into newly synthesized proteins, followed by click chemistry reaction with biotin alkyne. Biotin-labeled proteins can be detected by western blot analysis. **d** Click-iT AHA assay in WT and SMA MNs (N = 4). ACTB was used as loading control. **e** Scheme of experimental design. 10DIV MNs were treated with 10 µM NAC or 50 mM pyruvate for 1 h. Cells were incubated with 1 μM puromycin for 30 min before analysis. **f**, **g** SUnSET assay after ROS modification in (**f**) WT MNs (N = 5) and (**g**) SMA MNs (N = 5). Blue circles represent data from WT and red squares represent SMA MNs. Two-tailed unpaired t-tests with Holm-Bonferroni correction for multiple comparisons were used to determine statistical significance. All bar graphs depict the mean ± S.D., n.s. *p* > 0.05, *p < 0.05, **p < 0.01, ***p < 0.001

Carbonylation of proteins can alter their conformation and hinder protein synthesis [23, 48]. Furthermore, as levels of ribosomes and proteins associated with translation are also changed in SMA according to our MS data, we measured protein synthesis efficiency with Surface sensing of translation (SUnSET) and Click-iT AHA assay in WT and SMA MNs. Indeed, we confirmed that SMA MNs show a reduced protein synthesis efficiency compared to WT ones (Fig. 3a-d). Based on these data, we hypothesized that the elevated ROS levels hinder protein synthesis, therefore, reducing elevated ROS might restore impaired protein synthesis in SMA MNs. To further understand the effect of ROS on protein synthesis, we modified cellular ROS levels and measured protein synthesis efficiency. We treated MNs with 10 µM NAC, 50 mM pyruvate or 100 µM menadione and performed SUnSET analysis (Scheme in Fig. 3e). While protein synthesis efficiency was unaltered by pyruvate or NAC, menadione-induced ROS clearly inhibited protein synthesis in NSC-34 cells and WT MNs. As a negative control, 50 µM of the translation inhibitor anisomycin was used (Fig. 3f and Additional file 3: Figure S3a, b). Interestingly, NAC treatment could increase protein synthesis in SMA MNs, where cellular ROS levels are higher (Fig. 3g). However, pyruvate treatment failed to increase protein synthesis in SMA MNs (Fig. 3g). Taken together, our data strongly suggests that intracellular ROS levels influence protein synthesis in MNs.

### ROS regulates axonal local translation in MNs

**Fig. 4:**
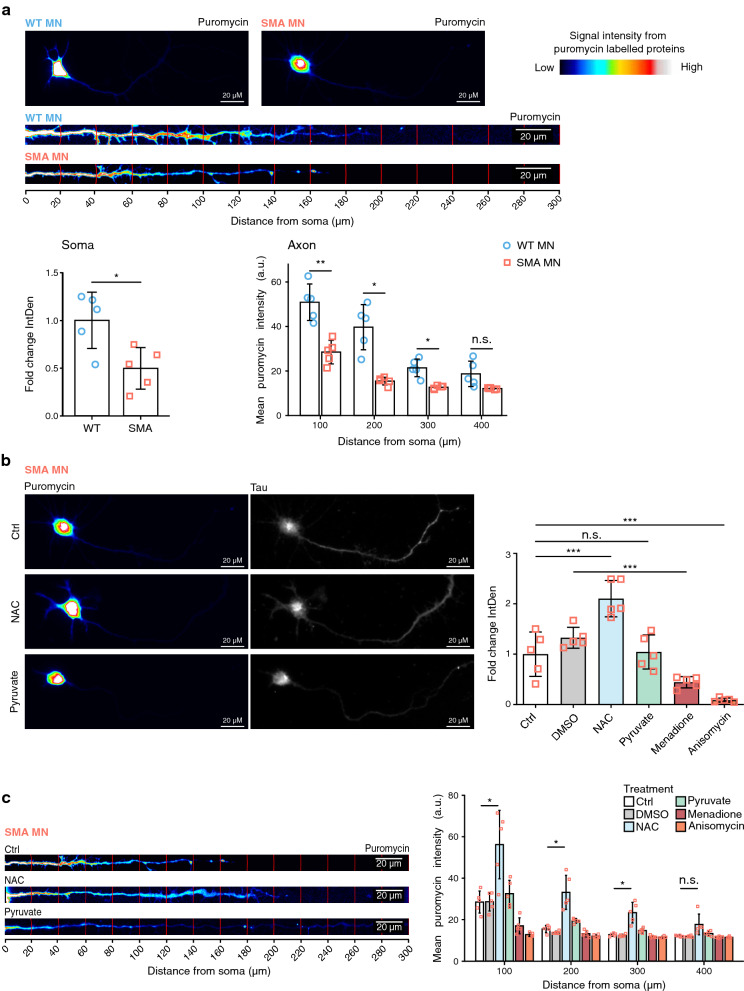
ROS regulates axonal local translation in MNs. **a** SUnSET assay. Representative images of WT and SMA MNs. Neurites were selected with a segmented line, straightened, and divided into 20 µm bins using the concentric circles plugin. Quantification of mean puromycin intensity profiles confirms that protein synthesis is reduced in the soma and axonal compartment of SMA MNs. **b** SUnSET assay. Representative images of SMA MNs after ROS modification. Tau staining shows whole neuronal morphology and puromycin signal represents newly synthesized proteins after 1 h treatment. 10 µM NAC, 100 µM menadione or 50 µM anisomycin were treated. Each dot represents the average intensity of 15 neurons (N = 5). One-way ANOVA with Tukey HSD post hoc analysis was used to determine statistical significance for multiple comparisons. Bar graphs depict the mean ± S.D. n.s. *p* > 0.05, ***p < 0.001. **c** SUnSET assay. Representative images of SMA axons. MNs were treated with 10 µM NAC, 50 mM pyruvate, 100 µM menadione, or 50 µM anisomycin for 1 h. **a–c** Intensity of incorporated puromycin signal is represented in rainbow scale. Scale bar: 20 µm. **a**, **c** Each dot represents the average quantification of 10 neurons of 5 individual biological replicates (N = 5). Bar graphs depict the mean ± S.D. Two-tailed unpaired t-test with Holm-Bonferroni correction for multiple comparisons was used to determine statistical significance. n.s. *p* > 0.05, *p < 0.05, **p < 0.01

Next, we investigated whether ROS regulates axonal local protein synthesis in MNs. We performed a SUnSET assay, but MNs were analyzed individually through imaging analysis. We are aware that local protein synthesis is lower in 10DIV-cultured MNs than in actively growing neurons [49, 50]. Therefore, we examined newly synthesized proteins in different axonal compartments. First, we measured anti-puromycin signals in 20 µm fractions and analyzed 100 µm fractions starting from the soma, comparing WT and SMA MNs. As expected, proximal fractions have higher anti-puromycin signals compared to distal parts. Importantly, the anti-puromycin signal in both soma and axonal part is lower in SMA MNs compared to WT ones (Fig. 4a). Next, we treated SMA MNs with NAC, pyruvate, menadione, and anisomycin and performed the same experiment. As expected, while NAC treatment increased protein synthesis, menadione or anisomycin inhibited protein synthesis in the soma as well as in the axonal compartment of SMA MNs (Fig. 4b, c and Additional file 3: Figure S4a). Consistent with the results in Fig. 3f, WT MNs did not show clear differences following NAC treatment (Additional file 3: Figure S4a and b).

### Whole proteome analysis of MNs with ROS manipulation

**Fig. 5:**
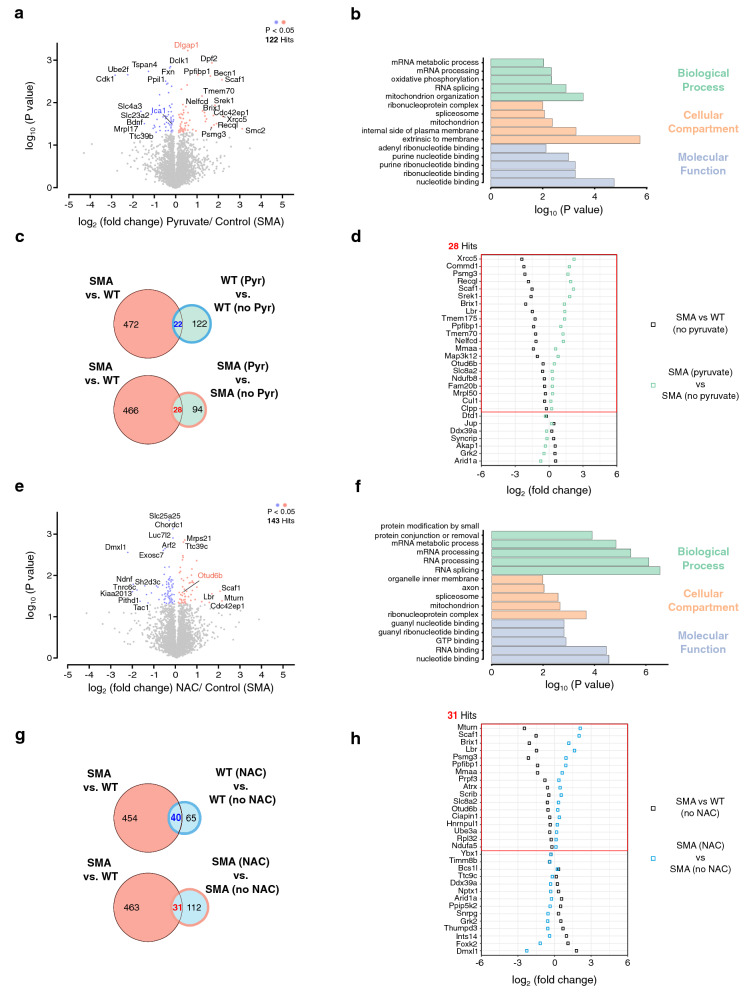
NAC and pyruvate modify the proteome of SMA MNs. **a**, **e** Volcano plot of whole proteome analysis in SMA MNs after (**a**) pyruvate or (**e**) NAC treatment; plotted *p* value (-log_10_) against fold change (log_2_) (N = 3). *p* values were determined by unpaired two-sided t-test. Significantly changed proteins with *p* < 0.05 are highlighted in blue (down-regulated) or red (up-regulated). **b, f** GO analysis of significantly changed proteins by (**b**) pyruvate or (**f**) NAC-treated SMA MNs. **c, g** Venn diagram showing the overlap of ‘significantly altered proteins in SMA compared to WT’ and ‘significantly changed proteins by (**c**) pyruvate or (**g**) NAC’ treatment in WT MNs or SMA MNs. **d** Levels of proteins altered in SMA compared to WT and by (**d**) pyruvate or (**h**) NAC treatment in SMA; plotted fold change (log_2_) comparing with and without pyruvate treatment

Next, we tried to obtain a systemic view of the whole proteome regulated by ROS in MNs. First, cells were incubated with 50 mM pyruvate for 1 h and the whole proteome was analyzed by MS. We found that the levels of 122 proteins were altered in SMA MNs (Fig. 5a). GO analysis of altered proteins suggests that pyruvate regulates proteins related to mitochondria (Fig. 5b). The biological process of oxidative phosphorylation was enriched, and the cellular compartment of mitochondria was affected by pyruvate supplementation (Fig. 5b). In addition, molecular functions involved in ribonucleotide binding, cellular compartments of the spliceosome, or biological processes such as RNA splicing and mRNA processing were also affected by pyruvate. Interestingly, these terms have previously been reported as altered pathways in SMA. Among the 494 proteins significantly changed in SMA MNs compared to WT MNs, 22 were also significantly changed by pyruvate in WT MNs, and 28 were significantly altered in pyruvate-treated SMA MNs (Fig. 5c). Intriguingly, when we compare proteins altered in SMA to pyruvate-affected proteins in WT or SMA MNs, we found that the effect of pyruvate was more apparent in SMA MNs (Fig. 5d and Additional file 3: Figure S5a). Among these 28 proteins, 21 proteins were down-regulated in SMA compared to WT and up-regulated by pyruvate in SMA (Fig. 5d). In contrast, pyruvate changed levels of 144 proteins in WT MNs, and among those, 22 proteins were also altered in SMA compared to WT (Fig. 5c and Additional file 3: Figure S5a, b). However, these 22 proteins showed far fewer changes after pyruvate treatment (Additional file 3: Figure S5a). Only two proteins, DLG-associated protein 1 (DLGAP1) and Islet cell autoantigen 1 (ICA1), were commonly changed by pyruvate treatment between WT and SMA MNs (Fig. 5a and Additional file 3: Figure S5b, c).

Subsequently, to obtain an overview of the antioxidant effect on the MN proteome, SMA MNs were treated with 10 µM NAC for 1 h. Whole proteome analysis identified that 143 proteins were significantly changed by NAC treatment (Fig. 5e). GO analysis suggested these proteins have multiple functions, including nucleotide-binding and RNA processing (Fig. 5f). Compared with differentially expressed proteins between WT and SMA MNs, 31 proteins were common with NAC treatment in SMA MNs (Fig. 5g). Among them, 17 proteins were down-regulated in SMA compared to WT, and up-regulated by NAC treatment in SMA (Fig. 5h). Again, NAC treatment showed little effect on WT MNs (Additional file 3: Figure S5d, e). NAC treatment altered only one protein, namely the deubiquitinase OTUD6B, in both WT and SMA MNs (Fig. 5e and Additional file 3: Figure S5e, f). ROS induction by 100 µM menadione for 1 h in WT MNs had the biggest effect on the proteome with 344 significantly altered proteins (Additional file 3: Figure S5g). In addition, menadione showed with 56 proteins the biggest overlap of altered proteins with proteins changed in SMA (Additional file 3: Figure S5h, i). It is worth mentioning that pyruvate and NAC had only a small effect on WT MNs, whereas they can induce a considerable up-regulation of proteins in SMA MNs. A possible explanation could be that the basal amounts of these proteins are lower in SMA MNs compared to WT MNs.

### ROS regulates initiation of mRNA translation

**Fig. 6:**
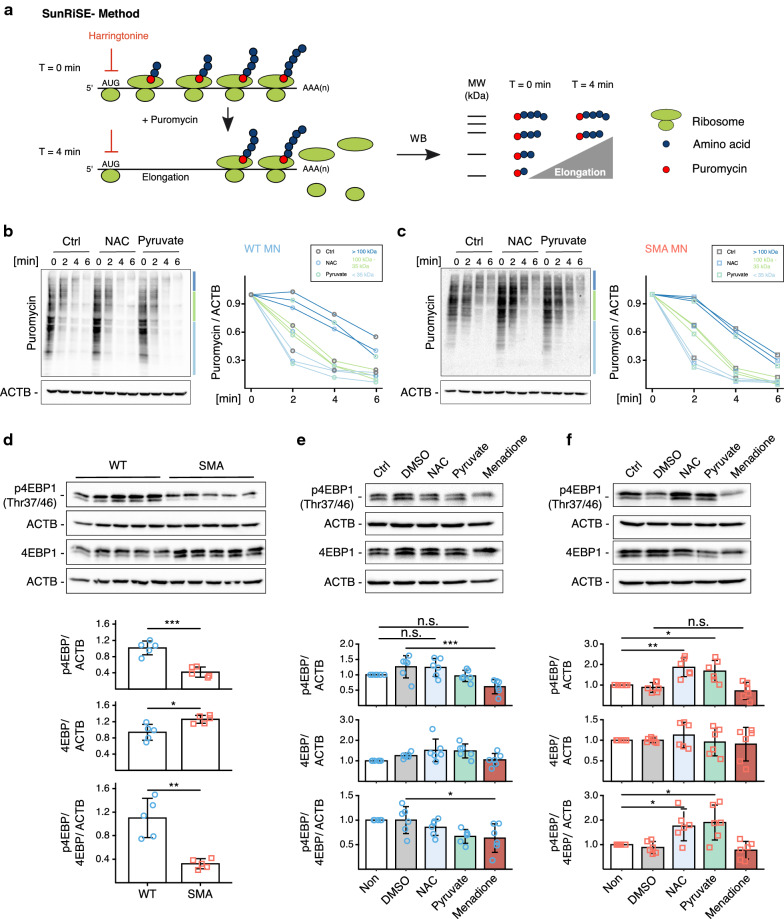
ROS regulates initiation of protein translation without altering elongation speed. **a** Scheme of SunRiSE assay. Translation initiation of 10DIV WT or SMA MNs was blocked with 2 µg/ml harringtonine at different time points before adding 10 μg/ml puromycin treatment for 10 min. **b, c** Neither NAC nor pyruvate changes elongation speed in (**b**) WT or (**c**) SMA MNs. Each dot and line represent the average of four independent biological replicates (N = 4). Regression analysis comparing the least square means does not show any significant difference between treatments. **d** Western blot analysis of p-4EBP1 and 4EBP1 levels in WT (blue circles) and SMA MNs (red squares) (N = 5). ATCB was used as loading control. Each dot represents the data from biological replicates. Two-tailed unpaired t-test was used to determine statistical significance *p < 0.05, **p < 0.01, ***p < 0.001. **e, f** Western blot analysis of p-4EBP1 and 4EBP1 levels after pharmacological modifications of ROS levels in (**e**) WT MNs (N = 6) and (**f**) SMA MNs (N = 6). Each dot represents the data from biological replicates. One-way ANOVA with Tukey HSD post hoc analysis was used to determine statistical significance for multiple comparisons. Bar graphs depict the mean ± S.D. n.s. *p* > 0.05, *p < 0.05, **p < 0.01, ***p < 0.001

Whole proteome analysis suggested that mRNA translation is dysregulated in SMA MNs (Fig. 1c). The volcano plot illustrates 360 proteins identified in our whole-proteome analysis related to mRNA translation based on the Mouse Genome Informatics (MGI) database (Additional file 3: Figure S6a). Among them, 40 out of 47 are significantly down-regulated in SMA MNs compared to WT (Additional file 3: Figure S6a). mRNA translation is tightly regulated in eukaryotic cells by two major processes: initiation and elongation. As it has already been reported that protein synthesis is impaired in SMA MNs [7, 8, 16], we aimed to seek further which step is disrupted by SMN loss. To distinguish the initiation and elongation of translation, we measured both processes in MNs. First, to assess the rate of protein elongation, we used the SunRiSE assay. In brief, translation initiation was blocked by 2 µg/ml harringtonine at different time intervals, then newly synthesized peptides were labeled with 10 µg/ml puromycin and detected by anti-puromycin antibody (Fig. 6a). The elongation speed was not altered by either NAC or pyruvate treatment in WT and SMA MNs (Fig. 6b, c). Furthermore, the elongation speed between WT and SMA MNs did not show any significant difference (Additional file 3: Figure S6b).

As translational elongation is unaltered in SMA or by ROS modification, we focused on translational initiation next. As one of the well-described translational initiation mechanisms is the cap-dependent translation initiation by 4E-BP1 (eukaryotic translation initiation factor 4E-binding protein 1), we measured the phosphorylation status of 4E-BP1 in WT and SMA MNs. A reduced phosphorylation status of 4E-BP1 indicates that initiation of mRNA translation is impaired in SMA MNs (Fig. 6d). Next, we measured the phosphorylation status in MNs after pharmacological modulation of ROS levels. While a significant difference was not observed by pyruvate or NAC treatment in WT cells, menadione impaired phosphorylation of 4E-BP1. This data suggests that excessive ROS can inhibit mRNA translation at the initiation step (Fig. 6e). Notably, pyruvate and NAC treatment increased the phosphorylation of 4E-BP1 in SMA MNs (Fig. 6f). Taken together, this unprecedented data reveals that mRNA translation is impaired at the initiation step in SMA MNs, while elongation is unaltered. Moreover, the initiation of mRNA translation can be regulated by ROS via 4E-BP1.

### SMN protein levels are regulated by pyruvate and ROS via mTOR

**Fig. 7:**
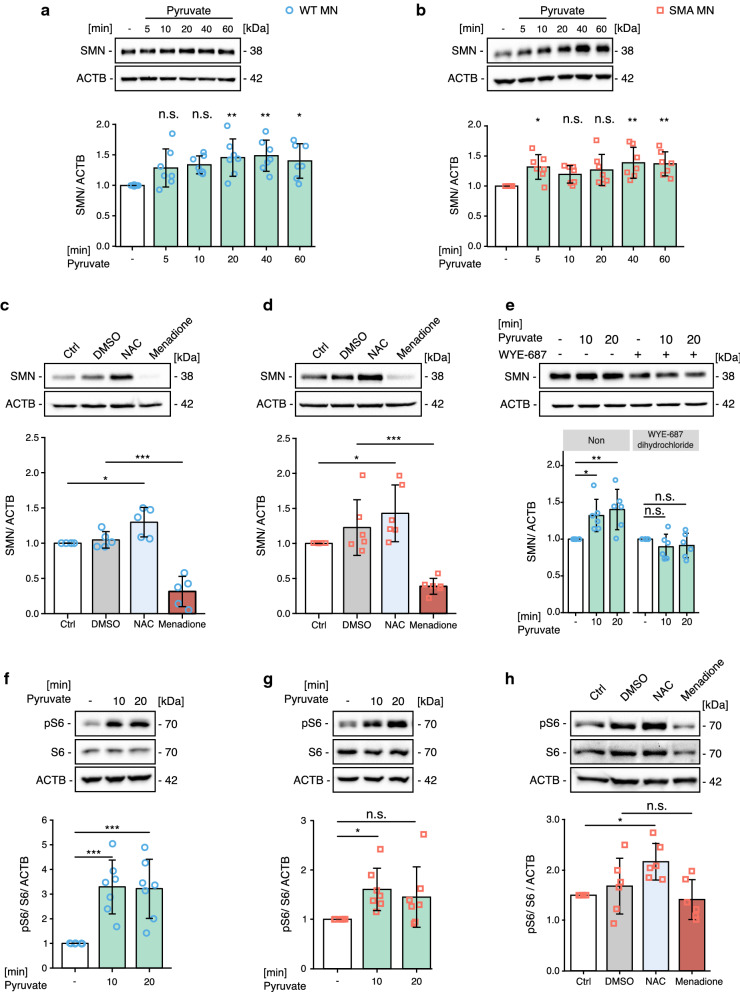
SMN levels are regulated by pyruvate and ROS modifying drugs. **a–d** Representative western blot images and quantification of SMN levels after pyruvate treatment for 1 h in (**a**) WT MNs and (**b**) SMA MNs or after ROS modification in (**c**) WT MNs and (**d**) SMA MNs. **e** 100 nM WYE-687 dihydrochloride blocks elevation of SMN levels after pyruvate treatment in WT MNs. **f–h** Representative western blot images and quantification of phosphorylated S6 and total S6 protein levels after pyruvate treatment in (**f**) WT MNs (N = 7) and (**g**) SMA MNs (N = 7) or after (**h**) NAC and menadione treatment in SMA MNs (N = 4). One-way ANOVA with Dunnett post hoc analysis was used to compare each time point with the control (**a**, **b**). One-way ANOVA with Tukey HSD post hoc analysis was used to determine statistical significance for multiple comparisons (**c**-**h**). Each dot represents the quantification of individual biological replicates. Bar graphs depict the mean ± S.D., n.s. *p* > 0.05, *p < 0.05, **p < 0.01, ***p < 0.001

Most interestingly, pyruvate increased SMN levels in WT and SMA MNs as well as NSC-34 cells (Fig. 7a, b, and Additional file 3: Figure S7a). To identify the molecular mechanism underlying increased SMN levels, we measured *Smn* mRNA levels in WT MNs and NSC-34 cells after pyruvate treatment and found no significant increase of *Smn* mRNA levels (Additional file 3: Figure S7b). This data suggests a post-transcriptional regulation of SMN levels. Next, we found that the protein synthesis inhibitor anisomycin could prevent pyruvate-induced elevation of SMN levels (Additional file 3: Figure S7c). This data confirms that protein synthesis of SMN is regulated by pyruvate post-transcriptionally. In addition, NAC increased SMN levels in MNs (Fig. 7c, d). As mTOR, especially mTORC1, is a central regulator of protein synthesis [51], we tested whether the increase of SMN levels is mTOR-dependent. We treated MNs with the water-soluble mTOR inhibitor WYE-687 dihydrochloride and subsequently with pyruvate. Indeed, pyruvate failed to increase SMN levels when mTOR activity was inhibited (Fig. 7e). In addition, pyruvate increased mTORC1 activity in WT MNs (Fig. 7f, g and Additional file 3: Figure S7d). Furthermore, a reduction of ROS by NAC treatment in SMA MNs increased the mTORC1 activity (Fig. 7h). Taken together, our data showed that cellular ROS and ATP levels regulate SMN protein synthesis via regulating mTORC1. Furthermore, re-balancing ROS levels with an antioxidant in SMA MNs can increase SMN protein synthesis.

## Discussion

Through whole proteome analysis, we identified that SMA MNs exhibit mitochondrial dysfunction. Prior studies have noted mitochondrial defects in SMA, including decreased respiration, increased oxidative stress, and impaired mitochondrial mobility [18, 20]. Here, we further investigated the molecular mechanisms underlying mitochondrial defects and reported that the function of complex I of the electron transport chain in mitochondria is impaired in SMA MNs. Furthermore, due to the dysfunctional complex I, intracellular ATP concentration is lower, and ROS levels are higher in SMA MNs. Regarding ROS generation, complex I and complex III are the known critical components. However, as the physiological or pathological relevance of complex III in ROS generation seems controversial, we focused on complex I function [22]. Moreover, complex I activity was not altered in the heart, another energy-demanding tissue. This finding indicates that complex I dysfunction can be a neuron-specific phenotype in SMA.

While a previous report did not show changes in the number of axonal mitochondria [20], we found that the number of axonal mitochondria is reduced. This controversy might be explained by methodological differences. In the previous study, mitochondria were labeled by aberrantly expressed mitochondrial proteins, while we used MitoTracker, a membrane potential dependent dye, which does not stain non-functional mitochondria or antibodies against the mitochondrial protein TOM20. This implicates that the proportion of dysfunctional mitochondria in SMA axons is higher compared to WT ones.

It has widely been accepted that neurons use mitochondria as their primary source of energy. However, a recent study suggested that neurons can obtain energy from glycolysis in energy-demanding conditions and locations [52]. Based on our results, glucose uptake is impaired in SMA MNs. Therefore, reduced energy production due to defective mitochondria might not be compensated by glycolysis. Taken together, we could conclude that SMA MNs are in a status of energy deprivation caused by mitochondrial defects as well as impaired glucose uptake. Glycolysis is also impaired in other neurodegenerative diseases such as AD and amyotrophic lateral sclerosis (ALS) [53, 54]. Furthermore, it has been proposed that enhancing glycolysis is neuroprotective in ALS [55]. However, it is unclear whether glycolysis is also impaired in SMA MNs. This needs to be investigated further.

As excessive cellular ROS are harmful to the cellular system, production and scavenging mechanisms are tightly regulated in all living organisms. Imbalanced ROS has been reported in numerous pathological conditions [56, 57]. ROS can be produced by mitochondria and cleared up by enzymes and chelating agents [58]. With our current data, we can speculate that ROS production can be increased by defective mitochondrial complex I, yet it is not clear whether ROS scavenging mechanisms are also altered in SMA MNs. Importantly, we revealed that ROS regulates mRNA translation at the initiation step and this mechanism is dysregulated in SMA. Counteracting this molecular pathway by supplementing pyruvate or the antioxidant NAC restores impaired translational initiation in SMA MNs. Nevertheless, in WT MNs, reducing ROS did not affect protein synthesis as WT MNs do not suffer from oxidative stress. This data intensifies our conclusion that healthy cells tightly regulate the balance of ROS levels, but SMA cells lost their ability to regulate ROS homeostasis due to defective mitochondrial complex I. Furthermore, our data revealed that ROS regulates mRNA translation at the initiation step, contributing to impaired protein synthesis, a known SMA pathology [16, 17]. Additionally, we found that the number and functionality of axonal mitochondria are reduced in SMA MNs. Therefore, to test whether impaired axonal mitochondria also influence protein synthesis in axons, we measured mRNA translation in the axonal compartment of the neurons. In a compartment-specific analysis, we revealed reduced protein synthesis in SMA MNs within the soma and along the axon. In 10 days cultured MNs, active protein synthesis was not observed at the distal part of the axons, while active protein synthesis could be observed and quantified in the proximal part of the axons.

Interestingly, reduction of ROS via pyruvate or NAC led to an increase of SMN protein levels in an mTOR-dependent manner. An increase of SMN levels by pyruvate or NAC was also observed in WT MNs, even though it did not change the overall protein synthesis rate. While the underlying molecular mechanism is unclear, our data suggests that pyruvate and NAC specifically increase SMN protein levels via the mTOR pathway without altering the whole proteome, independent of oxidative stress. It has previously been reported that SMN is involved in ribosome biology, and mTOR activity is reduced in SMA neurons [16, 17]. These findings suggest an SMN-specific feedback mechanism of gene expression. However, the mechanism underlying mTOR-dependent SMN protein synthesis needs to be investigated.

Overall, we unraveled the previously unknown molecular connections between mitochondria and protein synthesis by impairing translation initiation in neurons. This dysfunction contributes to the pathology of SMA via decreased ATP and increased ROS levels.

## Conclusion

This study describes the previously unknown mechanism that defective mitochondria influence SMA pathology, especially impaired protein synthesis. With a proteomics approach using an SMA mouse model, we identified that mitochondrial respiratory complex I is dysfunctional in SMA MNs. Due to this, ATP levels are decreased and ROS levels are increased in SMA MNs. Furthermore, we could restore the homeostasis of cellular energy and the redox system in SMA MNs by supplementation of pyruvate. Modifying ROS levels influences protein synthesis at the translation initiation step, which is impaired in SMA MNs. In addition, pyruvate enhances SMN protein synthesis in an mTOR-dependent manner. As mitochondrial defects have been reported in other neurological disorders, our study reveals the basic cellular mechanism of how mitochondria can influence protein synthesis in MNs. This study will lead to new insights into pharmacologically targetable pathways for neurological disorders, including SMA.

## Supplementary Information

**Additional file 1:** Supplementary Table S1. Primer sequences. S2. Antibodies and conditions for Western blot (WB) and immunofluorescence (IF). S3. Fluorescence dyes. S4. Drugs and supplements for in vitro assays.

**Additional file 2:** Mass spectromertry source data. This file contains results of the mass spectrometry including pathway analyses.

**Additional file 3:** Supplementary Figure S1. Complex I deficiency leads to dysfunctional and fragmented mitochondria. S2. Mitochondrial complex I is impaired in SMA MNs. S3. Optimization of SUnSET assay. S4. Reduction of ROS improves protein synthesis in SMA MNs, but not in WT MNs. S5 Whole cell proteome after modifying ROS levels. S6 Proteins related to translation are significantly changed in SMA MNs without affecting elongation speed. S7. Pyruvate regulates SMN levels.

## Data Availability

The mass spectrometry proteomics data have been deposited to the ProteomeXchange Consortium via the PRIDE [59] partner repository with the dataset identifier PXD020403. The results of the mass spectrometry dataset, supporting the conclusions of this article is included within the article as Additional file 2: Mass Spectrometry source data.

## Abbreviations

4E-BP1: eukaryotic initiation factor 4E (eIF4E)- binding protein 1
ACTB: actin beta
AD: Alzheimer’s disease
ALS: amyotrophic lateral sclerosis
ANOVA: analysis of variance
ATP: adenosine triphosphate
Complex I: NADH:ubiquinone oxidoreductase
DIV: days in vitro
DLGAP1: DLG-associated protein 1
DMEM: Dulbecco’s Modified Eagle’s Medium
eIF4E: eukaryotic translation initiation factor 4E
GO: gene ontology
ICA1: islet cell autoantigen 1
MGI: Mouse Genome Informatics
MN: motor neuron
MS: mass spectrometry
mRNA: messenger ribonucleic acid
miRNA: micro ribonucleic acid
mTORC1: mammalian target of rapamycin complex 1
NAC: N-acetylcysteine
OXPHOS: oxidative phosphorylation
RNA: ribonucleic acid
ROS: reactive oxygen species
S6: ribosomal protein S6
S6K: S6 kinase
SDB-RPS: styrenedivinylbenzene-reverse phase sulfonate
SMA: spinal muscular atrophy
SMN: survival of motor neuron
SunRiSE: SUnSET-based Ribosome Speed of Elongation
SUnSET: surface sensing of translation
TCA: tricarboxylic acid
TSC: tuberous sclerosis complex
WT: wildtype
