# Supplementary material for: Mitochondrial defects in the respiratory complex I contribute to impaired translational initiation via ROS and energy homeostasis in SMA motor neurons
Source: Acta Neuropathol Commun. 2020 Dec 22;8:223. doi: 10.1186/s40478-020-01101-6 (PMC7754598; doi:10.1186/s40478-020-01101-6)
Supplement: Supplementary file 1 — Additional file 1: Supplementary Table S1. Primer sequences. S2. Antibodies and conditions for Western blot (WB) and immunofluorescence (IF). S3. Fluorescence dyes. S4. Drugs and supplements for in vitro assays. [file 40478_2020_1101_MOESM1_ESM.docx]

**Supplementary Table 1:** Primer sequences

| **Applications** | **Name** | **Sequence** | **Expected size** |
| --- | --- | --- | --- |
| qRT-PCR | *Actb*-fw | AGCCATGTACGTAGCCATCC | 201 |
|  | *Actb*-rev | CTCTCAGCTGTGGTGGTGAA |  |
|  | *Smn*-fw | ACTCCTCCAGATCGCTCAGA | 227 |
|  | *Smn*-rev | AGGGGGTGGCGGGATTATTG |  |

**Supplementary Table 2:** Antibodies and conditions for Western blot (WB) and immunofluorescence (IF)

| **Primary Antibodies** | **Host species/dilution** | **Manufacturer/ #catalog** | **RRID** |
| --- | --- | --- | --- |
| anti-4E-BP1 | rabbit; WB 1:1000 | Cell Signaling; #9644 | AB_2097841 |
| anti-p4E-BP1 (Thr37/46) | rabbit; WB 1:1000 | Cell Signaling; #2855 | AB_560835 |
| anti-ACTB, HRP-conjugated | mouse; WB 1:10,000 | Santa Cruz; sc-47778 HRP | AB_2714189 |
| anti-ChAT | rabbit; IF 1:100 | Thermo Scientific; PA5-26597 | AB_2544097 |
| anti-Puromycin | mouse; WB 1:1500  IF 1:150 | Merck; MABE343 | AB_2566826 |
| anti-S6 | mouse; WB 1:1000 | Cell Signaling; #2317 | AB_2238583 |
| anti-pS6 | rabbit; WB 1:1000 | Cell Signaling; #2211 | AB_331679 |
| anti-S6K | rabbit; WB 1:2000 | Cell Signaling; #2708 | AB_390722 |
| anti-pS6K | rabbit; WB 1:750 | Cell Signaling; #9234 | AB_2269803 |
| anti-SMN | mouse; WB 1:3000 | BD Biosciences; 610646 | AB_397973 |
| anti-Tau | mouse; IF 1:800 | Santa Cruz; sc-390476 |  |
| anti-Tau | chicken; IF 1:100 | Abcam; ab75714 | AB_1310734 |
| anti-TOM20 | rabbit; IF 1:500 | Santa Cruz; sc-11415 | AB_2207533 |

| **Secondary antibodies** | **Host species/dilution** | **Manufacturer/ #catalog** | **RRID** |
| --- | --- | --- | --- |
| anti-mouse IgG, HRP-conjugated | goat; WB 1:3000 | Dianova; 115-035-146 | AB_2307392 |
| anti-rabbit IgG, HRP-conjugated | goat; WB 1:2000 | Cell Signaling; #7074 | AB_2099233 |
| anti-chicken IgG, AlexaFluor647-conjugated | goat; IF 1:350 | Thermo Scientific; A21449 | AB_1500594 |
| anti-mouse IgG, AlexaFluor488-conjugated | goat; IF 1:350 | Thermo Scientific; A11001 | AB_2534069 |
| anti-rabbit IgG, AlexaFluor488-conjugated | donkey; IF 1:350 | Thermo Scientific; A21206 | AB_2535792 |
| anti-rabbit IgG, AlexaFluor568-conjugated | donkey; IF 1:350 | Thermo Scientific; A10042 | AB_2534017 |

**Supplementary Table 3:** Fluorescence dyes

| **Fluorescence dye** | **Manufacturer/ #catalog** |
| --- | --- |
| Alexa Fluor^TM^ 568 Phalloidin | Thermo Scientific; A12380 |
| CellROX^TM^ Green reagent | Thermo Scientific; C10444 |
| DAPI | Thermo Scientific; R37606 |
| MitoTracker^®^ Red CMXRos | Thermo Scientific; M7512 |

**Supplementary Table 4:** Drugs and supplements for *in vitro* assays

| **Drugs/ supplements** | **Concentration** | **Solvent** | **Manufacturer/ #catalog** |
| --- | --- | --- | --- |
| Anisomycin | 40 µM/ 50 µM | Water | Sigma; A9789 |
| Harringtonine | 2 mg/ml | DMSO | Abcam; ab141941 |
| Menadione | 0.1 µM-100 µM | DMSO | Sigma; M5625 |
| N-Acetyl-L-cysteine (NAC) | 1 µM-1000 µM | Water | Sigma; A9165 |
| Puromycin Dihydrochloride | 1 µM/ 10 µg/ml | Water | Gibco; A1113803 |
| Sodium pyruvate | 1 mM – 50 mM | Water | Sigma; P5280 |
| Sodium lactate | 1 mM – 50 mM | Water | Sigma; L7022 |
| WYE-687 dihydrochloride | 100 nM | Water | Tocris; #4282 |
