# Supplementary material for: Mitochondrial defects in the respiratory complex I contribute to impaired translational initiation via ROS and energy homeostasis in SMA motor neurons
Source: Acta Neuropathol Commun. 2020 Dec 22;8:223. doi: 10.1186/s40478-020-01101-6 (PMC7754598; doi:10.1186/s40478-020-01101-6)
Supplement: Supplementary file 3 — Additional file 3: Supplementary Figure S1. Complex I deficiency leads to dysfunctional and fragmented mitochondria. S2. Mitochondrial complex I is impaired in SMA MNs. S3. Optimization of SUnSET assay. S4. Reduction of ROS improves protein synthesis in SMA MNs, but not in WT MNs. S5 Whole cell proteome after modifying ROS levels. S6 Proteins related to translation are significantly changed in SMA MNs without affecting elongation speed. S7. Pyruvate regulates SMN levels. [file 40478_2020_1101_MOESM3_ESM.pdf]

Figure S1

a

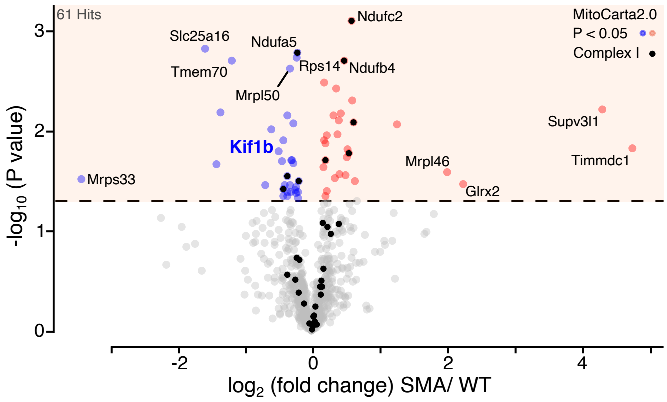

• Complex I Hits	P value	Fold change
Ndufb8	0,038	0,75
Ndufv2	0,028	0,78
Ndufa5	0,002	0,86
Ndufb5	0,032	0,88
Ndufa6	0,008	1,55
Ndufc2	0,001	1,52
Ndufab1	0,017	1,47
Ndufb4	0,002	1,40
Ndufa10	0,019	1,16

b

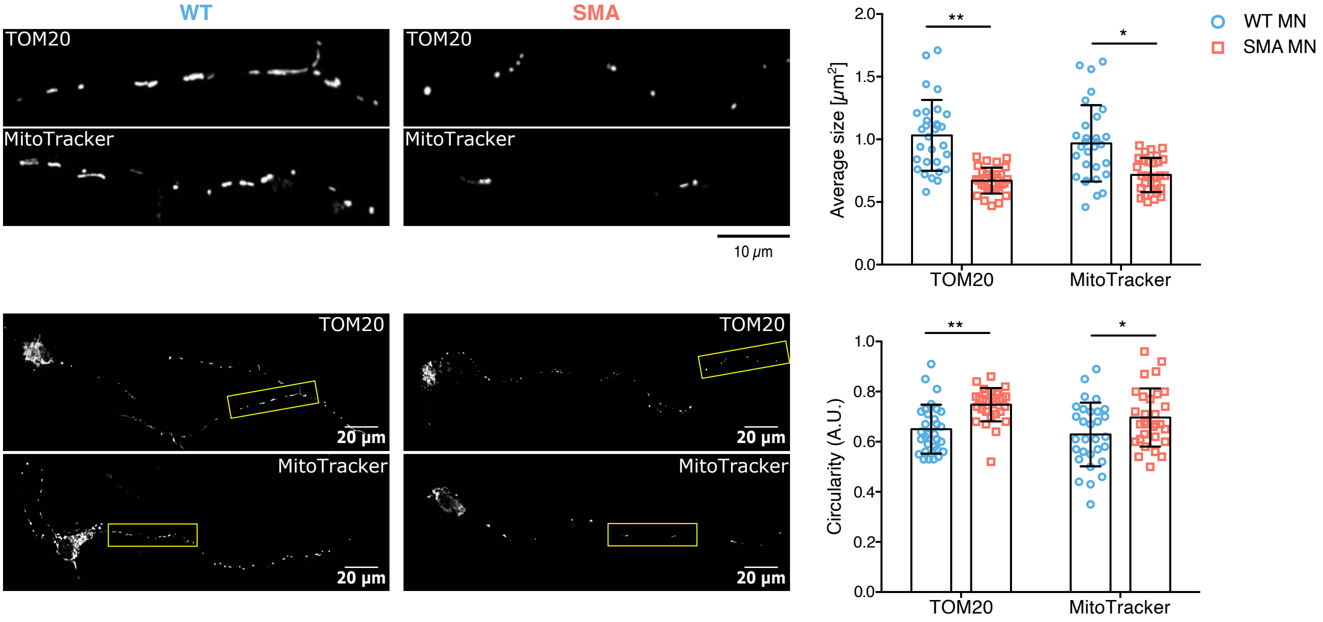

Fig.S1 Complex I deficiency leads to dysfunctional and fragmented mitochondria.

a Volcano plot of proteins identified in whole proteome analysis and reported in the MitoCarta2.0 database; plotted p-values (-log_{10}) against fold changes (log_2, SMA/ WT). Four independent samples of WT MNs and three independent samples of SMA MNs were used for analysis. P-values were determined using unpaired two-sided t-test. Proteins with $p < 0.05$ are highlighted in blue (32 down-regulated) or red (29 up-regulated).

b Representative images and quantification of mitochondrial size and circularity of 10DIV WT and SMA MNs labelled with anti-TOM20 antibody or MitoTracker®. Enlarged areas are highlighted with yellow boxes in the corresponding image of the whole neuron. Scale bar in enlarged images: 10μm; Scale bar in whole neuron images: 20μm. Each dot represents the quantification of individual neurons (n=30). To compare WT (blue circles) and SMA (red squares), two-way ANOVA with Tukey HSD post hoc analysis was used on independent biological replicates (N=3) to determine statistical significance. Bar graphs depict the mean ± s.d. *$p < 0.05$, **$p < 0.01$.

Figure S2

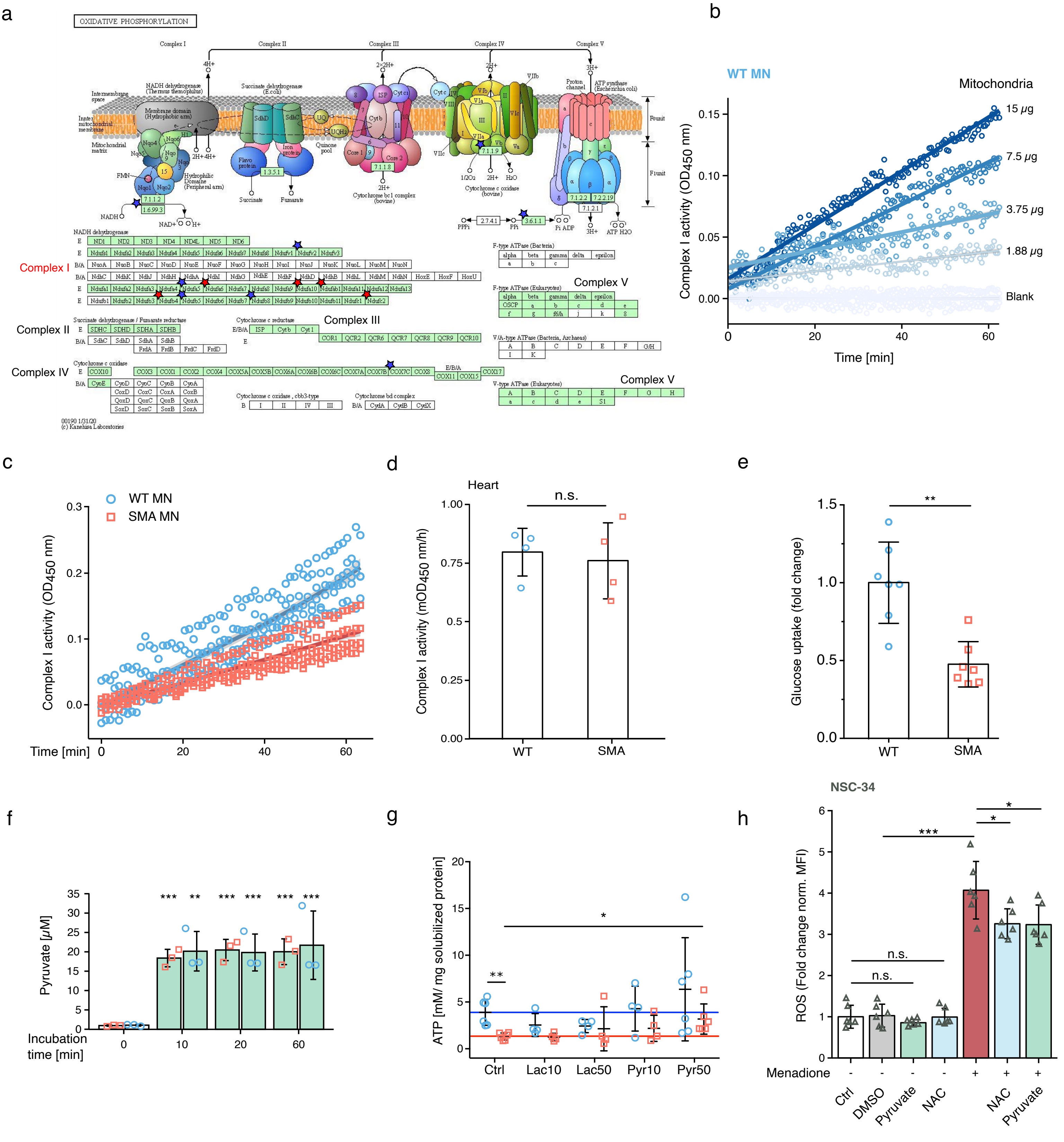
a
OXIDATIVE PHOSPHORYLATION
Complex I
Complex II
Complex III
Complex IV
Complex V
NADH dehydrogenase
Succinate dehydrogenase / Fumarate reductase
Cytochrome c reductase
Cytochrome c oxidase
F-type ATPase (Bacteria)
F-type ATPase (Eukaryotes)
V/A-type ATPase (Bacteria, Archaeas)
V-type ATPase (Eukaryotes)
Cytochrome c oxidase, cbb3-type
Cytochrome bd complex
00190 1/31/20
(c) Kanehisa Laboratories
b
WT MN
Mitochondria
15 µg
7.5 µg
3.75 µg
1.88 µg
Blank
Complex I activity (OD450 nm)
Time [min]
c
WT MN
SMA MN
Complex I activity (OD450 nm)
Time [min]
d
Heart
n.s.
Complex I activity (mOD450 nm/h)
WT
SMA
e
**
Glucose uptake (fold change)
WT
SMA
f
*** ** *** *** *** ***
Pyruvate [µM]
Incubation time [min]
0
10
20
60
g
**
*
ATP [mM/ mg solubilized protein]
Ctrl
Lac10
Lac50
Pyr10
Pyr50
h
NSC-34
***
*
*
n.s.
n.s.
ROS (Fold change norm. MFI)
Menadione
- - - - + + +
Ctrl
DMSO
Pyruvate
NAC
NAC
Pyruvate

Fig.S2 Mitochondrial complex I is impaired in SMA MNs.

a Representation of oxidative phosphorylation KEGG Pathway (mmu00190) [1]. Green boxes highlight organism-specific complexes (Mus musculus). Stars mark significantly up-regulated (red) and down-regulated (blue) proteins in SMA MNs (10DIV).

b Optimization of Complex I activity using different amounts of mitochondria extract isolated from 10DIV WT MNs. Linear regression shows that complex I activity and reaction time is proportional over 1 h.

c Complex I activity using 20 μg mitochondria extract isolated from 10DIV WT and SMA MNs. Linear regression shows that complex I activitiy from WT and SMA MNs is proportional over 1 h, with a reduced slope for SMA MNs.

d Complex I activity rate using 300 μg protein extracts of heart from P7 WT and SMA mice (N=4). Quantification represents the increase of mean OD_{450} nm/ h. Two-way ANOVA with Tukey HSD post hoc analysis was used on independent biological replicates (N=4) to determine statistical significance. n.s. p >0.05.

e Glucose uptake in 10DIV WT and SMA MNs. Each dot represents data from biological replicates (N=7). Two-tailed unpaired t-test was used to determine statistical significance. **p <0.01.

f Pyruvate uptake is increased in WT MNs and SMA after 1 h supplementation (N=3). One-way ANOVA with Dunnett post hoc analysis was used to compare each timepoint with the control. **p <0.01, ***p<0.001.

g Supplementation of WT MNs and SMA MNs with 10 mM/ 50 mM lactate or 10mM/ 50 mM pyruvate for 1 h. 50 mM pyruvate treatment shows a significant increase of ATP levels in SMA MNs (N=6). Two-tailed unpaired t-test was used on independent biological replicates to determine statistical significance. Means of two groups were compared, *p <0.05, **p <0.01.

h Quantification of mean fluorescence intensity of CellROX signal in NSC-34 cells. One hour treatment of 100 μM menadione increases ROS levels, whereas co-treatment of 10 μM NAC or 50 mM reduces menadione-induced ROS (N=6). One-way ANOVA with Tukey HSD post hoc analysis was used on independent biological replicates (N=3) to determine statistical significance. n.s. p >0.05, *p <0.05, ***p <0.001.

Bar graphs scatterplots depict the mean ± s.d.

1. Kanehisa M (2000) KEGG: Kyoto Encyclopedia of Genes and Genomes. Nucleic Acids Research 28:27–30. doi: 10.1093/nar/28.1.27

Figure S3

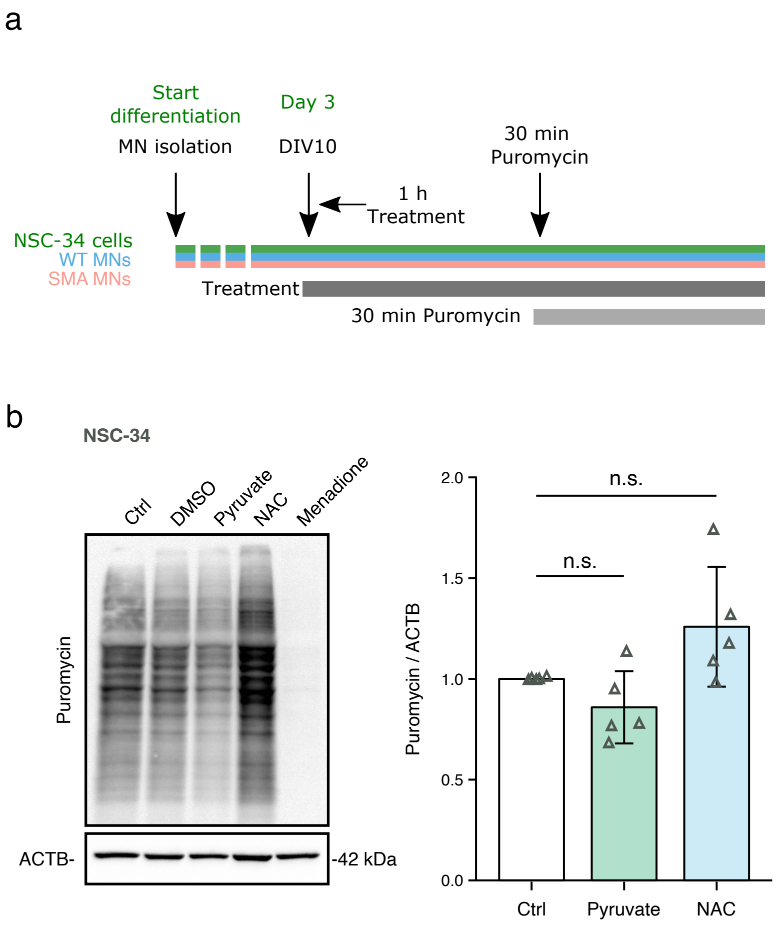

Fig.S3 Optimization of SUnSET assay

a Schematic representation of SUnSET assay in NSC-34 cells, WT MNs and SMA MNs. NSC-34 cells are differentiated for 3 days and MNs are cultured for 10 days before treatment with 50 mM pyruvate, 10 μM NAC or 100 μM menadione for 1 h. Puromycin was added in addition after 30 min of treatment.

b Representative western blot images and quantification of SUnSET assay in NSC-34 cells. Neither 50 mM pyruvate nor 10 μM NAC increased protein synthesis significantly. Each dot represents the quantification of individual biological replicates (N=5). Bar graphs depict the mean ± s.d. Two-tailed unpaired t-test with Holm-Bonferroni correction for multiple comparisons was used to determine statistical significance. n.s. p >0.05.

Figure S4

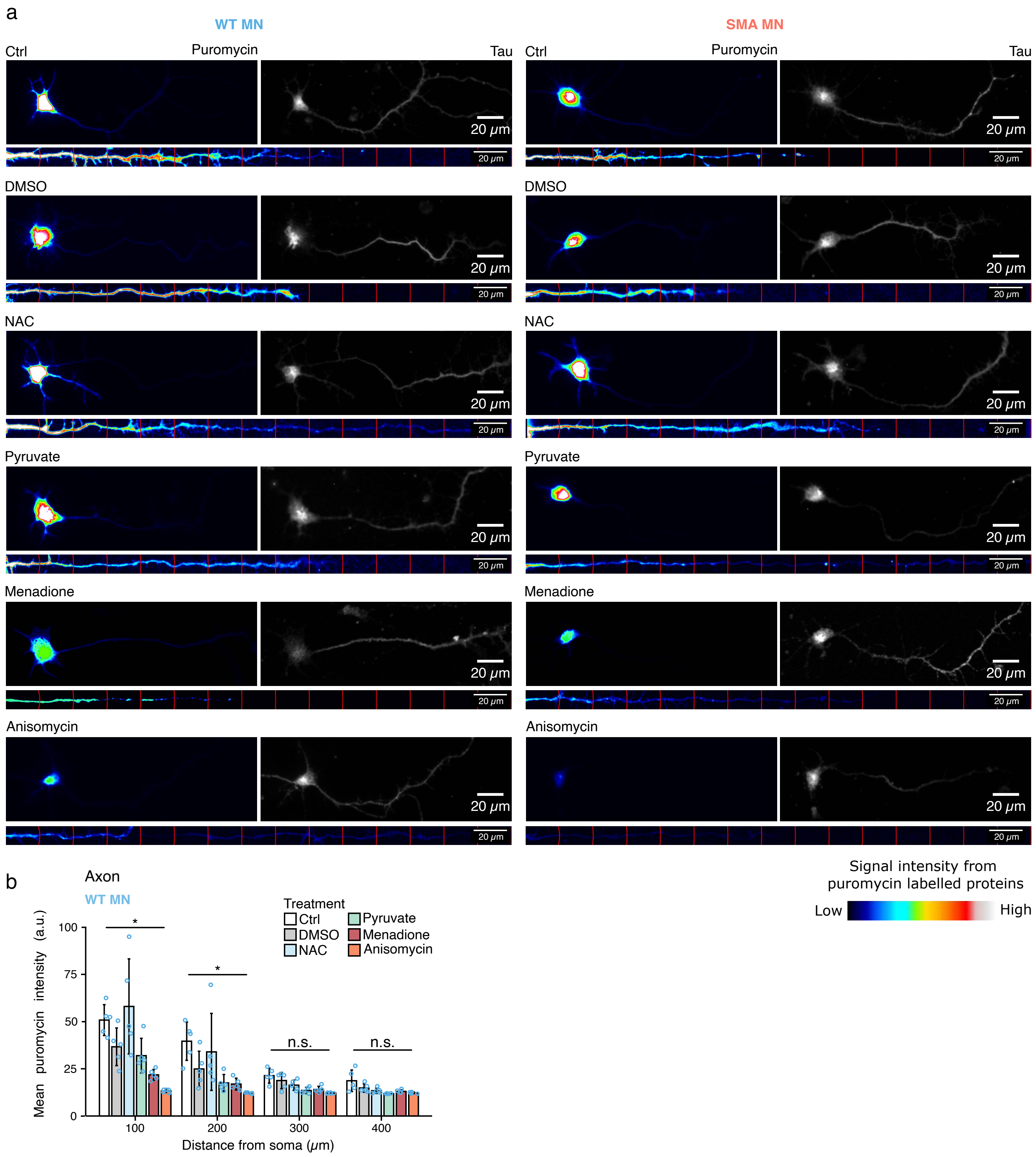

Fig.S4 Reduction of ROS improves protein synthesis in SMA MNs, but not in WT MNs.

a Representative images of WT and SMA MNs after SUnSET assay. Anti-puromycin (rainbow color) and anti-Tau (white) antibodies are used. Tau positive neurites (axons) were selected with a segmented line, straightened and divided into 20 μm bins using the concentric circles plugin. MNs were treated with 10 μM NAC or 50 mM pyruvate or 100 μM menadione or 50 μM anisomycin for 1 h. Images confirm that protein synthesis is blocked by anisomycin or menadione and increased in SMA MNs by 10 μM NAC. Scale bar: 20 μm.

b Quantification of mean puromycin intensity profiles, corresponding to protein levels, against distance in discrete categories. Each dot represents the average quantification of 10 neurons. Data are obtained from 5 individual biological replicates (N=5). Bar graph depict the mean ± s.d. Two-tailed unpaired t-test with Holm-Bonferroni correction for multiple comparisons was used to determine statistical significance. n.s. $p > 0.05$, *$p < 0.05$.

Figure S5

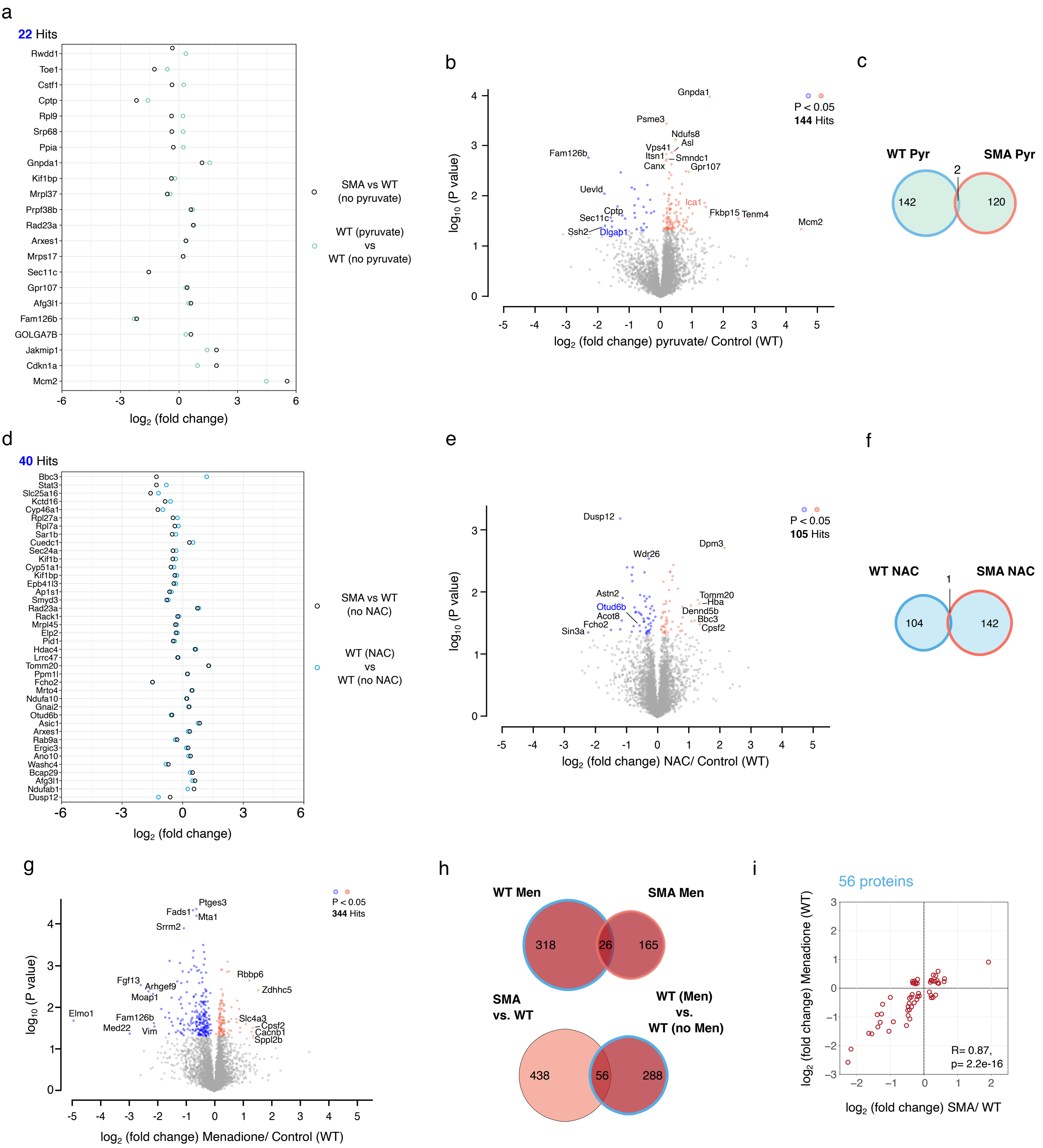

a
22 Hits
Rwdd1
Toe1
Cstf1
Cptp
Rpl9
Srp68
Ppia
Gnpda1
Kif1bp
Mrpl37
Prpf38b
Rad23a
Arxes1
Mrps17
Sec11c
Gpr107
Afg3l1
Fam126b
GOLGA7B
Jakmip1
Cdkn1a
Mcm2
-6
-3
0
3
6
log2 (fold change)
SMA vs WT (no pyruvate)
WT (pyruvate) vs WT (no pyruvate)
b
Gnpda1
P < 0.05
144 Hits
Psme3
Ndufs8
Asl
Fam126b
Vps41
Itsn1
Smndc1
Canx
Gpr107
Uevld
Ica1
Cptp
Fkbp15
Tenm4
Sec11c
Mcm2
Ssh2
Dlgap1
log10 (P value)
log2 (fold change) pyruvate/ Control (WT)
c
WT Pyr
SMA Pyr
2
142
120
d
40 Hits
Bbc3
Stat3
Slc25a16
Kctd16
Cyp46a1
Rpl27a
Rpl7a
Sar1b
Cuedc1
Sec24a
Kif1b
Cyp51a1
Kif1bp
Epb41l3
Ap1s1
Smyd3
Rad23a
Rack1
Mrpl45
Elp2
Pid1
Hdac4
Lrrc47
Tomm20
Ppm1l
Fcho2
Mrto4
Ndufa10
Gnai2
Otud6b
Asic1
Arxes1
Rab9a
Ergic3
Ano10
Washc4
Bcap29
Afg3l1
Ndufab1
Dusp12
log2 (fold change)
SMA vs WT (no NAC)
WT (NAC) vs WT (no NAC)
e
Dusp12
P < 0.05
105 Hits
Dpm3
Wdr26
Astn2
Tomm20
Hba
Otud6b
Dennd5b
Acot8
Bbc3
Fcho2
Cpsf2
Sin3a
log10 (P value)
log2 (fold change) NAC/ Control (WT)
f
WT NAC
SMA NAC
1
104
142
g
Ptges3
P < 0.05
344 Hits
Fads1
Mta1
Srrm2
Rbbp6
Fgf13
Zdhhc5
Arhgef9
Moap1
Elmo1
Fam126b
Slc4a3
Cpsf2
Med22
Vim
Cacnb1
Sppl2b
log10 (P value)
log2 (fold change) Menadione/ Control (WT)
h
WT Men
SMA Men
318
26
165
SMA vs. WT
WT (Men) vs. WT (no Men)
438
56
288
i
56 proteins
log2 (fold change) Menadione (WT)
R= 0.87,
p= 2.2e-16
log2 (fold change) SMA/ WT

Fig.S5 Whole cell proteome after modifying ROS levels.

a Graph representing 22 proteins changed in SMA MNs compared to WT MNs, and pyruvate treated WT MNs compared to non-treated WT MNs; plotted fold change (log_2) comparing with and without pyruvate treatment.

b Volcano plot of whole proteome analysis after 50 mM pyruvate treatment to WT MNs for 1 h; plotted p-value ($-log_{10}$) against fold change (log_2). Four independent samples were used for analysis. P-values were determined using an unpaired two-sided t-test. Proteins with $p < 0.05$ are highlighted in blue (down-regulated) and red (up-regulated).

c Venn diagram showing quantity of proteins in WT MNs compared to SMA MNs treated with 50 mM pyruvate for 1 h.

d Graph representing 40 proteins changed in SMA MNs compared to WT MNs, and NAC treated WT MNs compared to non-treated WT MNs; plotted fold change (log_2) comparing with and without NAC treatment.

e Volcano plot of whole proteome analysis after 10 μM NAC treatment to WT MNs for 1 h; plotted p-value ($-log_{10}$) against fold change (log_2). Four independent samples were used for analysis. P-values were determined using an unpaired two-sided t-test. Proteins with $p < 0.05$ are highlighted in blue (down-regulated) and red (up-regulated).

f Venn diagram showing quantity of proteins in WT MNs compared to SMA MNs treated with 10 μM NAC for 1 h.

g Volcano plot of whole proteome analysis after 100 μM menadione to WT MNs for 1 h; plotted p-value ($-log_{10}$) against fold change (log_2). Four independent samples were used for analysis. P-values were determined using an unpaired two-sided t-test. Proteins with $p < 0.05$ are highlighted in blue (down-regulated) and red (up-regulated).

h Upper Venn diagram showing quantity of proteins in WT MNs compared to SMA MNs treated with 100 μM menadione for 1 h. Lower Venn diagram representing 56 proteins commonly altered in SMA and after menadione treatment.

i Scatterplot showing the fold changes (log_2) of significantly changed proteins in SMA against WT MNs after menadione treatment.

Figure S6

a

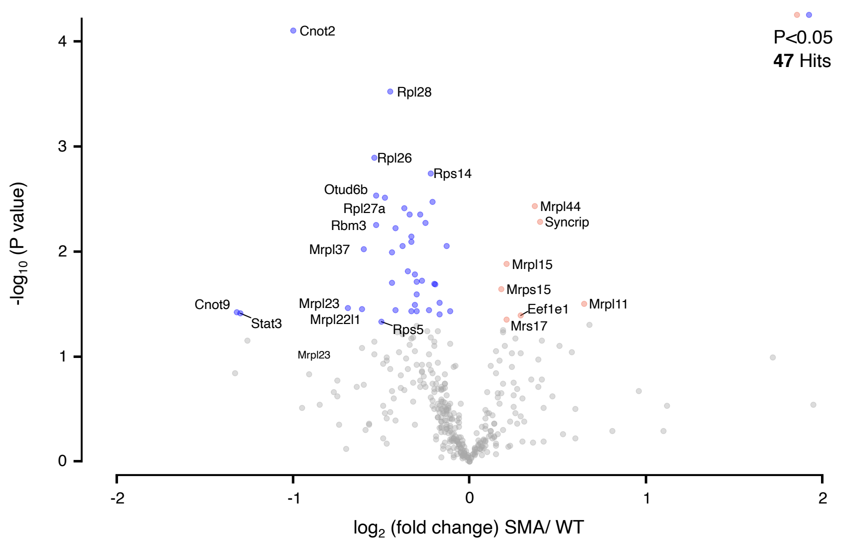

b

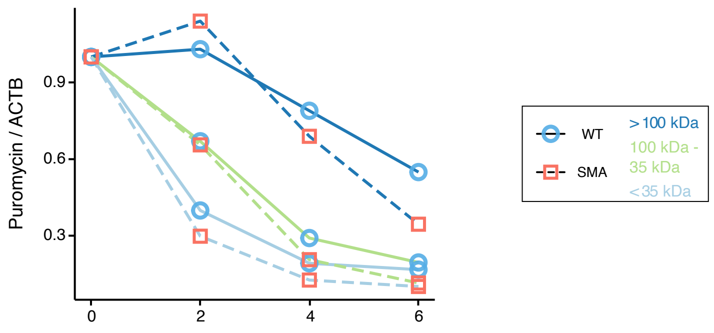

Fig.S6 Proteins related to translation are significantly changed in SMA MNs without affecting elongation speed.

a Volcano plot of translation related proteins comparing WT and SMA MNs; plotted p-value (-$\log_{10}$) against fold change ($\log_2$, SMA/ WT). Four independent samples of WT MNs and three independent samples for SMA MNs were used for analysis. P-values were determined using an unpaired two-sided t-test. Proteins with $p < 0.05$ are highlighted in blue (down-regulated) and red (up-regulated).

b Elongation speed is not altered in SMA MN compared to WT MNs. Each dot and each line represent the average of four independent biological replicates (N=4). Regression analysis comparing the least square means does not show any significant difference between WT MNs and SMA MNs.

Figure S7

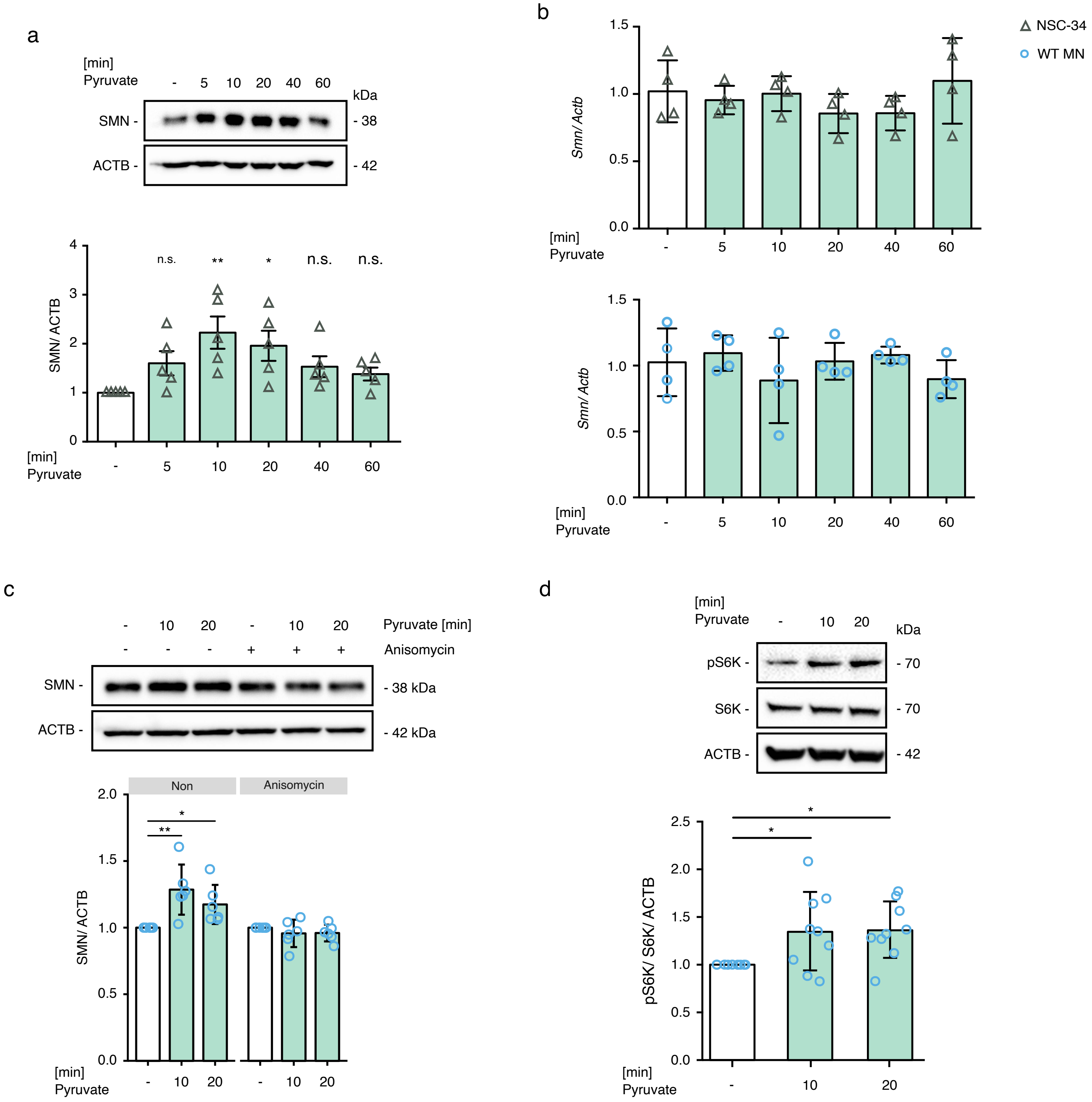

Fig.S7 Pyruvate regulates SMN levels.

a Representative western blot and quantification shows that 50 mM pyruvate increases SMN protein levels in NSC-34 cells after 1 h treatment (N=5). One-way ANOVA with Dunnett post hoc analysis was used to compare each timepoint with the control. n.s. $p>0.05$, *$p<0.05$, **$p<0.01$.

b Quantitative real-time PCR using gene specific Smn primers confirms that pyruvate supplementation did not change Smn transcript levels in NSC-34 cells and WT MNs (N=4). Actb was used as a loading control.

c 50 μM anisomycin prevents pyruvate-induced elevation of SMN protein levels (N=6). 50 mM pyruvate was treated for 10 or 20 min. One-way ANOVA with Tukey HSD post hoc analysis was used to determine statistical significance. *$p<0.05$, **$p<0.01$.

d Representative western blot and quantification show that pyruvate treatment increases phosphorylation status of p70S6 kinase. One-way ANOVA with Tukey HSD post hoc analysis was used to determine statistical significance. *$p<0.05$.
Each dot represents the quantification of individual biological replicates. Bar graph depict the mean ± s.d.
